# Supplementary material for: Dynamic computational phenotyping of human cognition
Source: Nat Hum Behav. 2024 Feb 8;8(5):917–31. doi: 10.1038/s41562-024-01814-x (PMC11132988; doi:10.1038/s41562-024-01814-x)
Supplement: Supplementary file 1 — Supplementary methods (experimental tasks and computational models), discussion, Table 1 and Figs. 1–18. [file 41562_2024_1814_MOESM1_ESM.pdf]

nature human behaviour

Article https://doi.org/10.1038/s41562-024-01814-x

Dynamic computational phenotyping of human cognition

In the format provided by the authors and unedited

Contents

1 Experimental tasks **2**

1.1 Go/No-Go 2

1.2 Change Detection 2

1.3 Random Dot Motion 3

1.4 Lottery Ticket 3

1.5 Intertemporal Choice 4

1.6 Two-armed Bandit 4

1.7 Numerosity Comparison 5

2 Computational models **6**

2.1 Go/No-Go 6

2.2 Change Detection 6

2.3 Random Dot Motion 8

2.4 Lottery Ticket 8

2.5 Intertemporal Choice 8

2.6 Two-Armed Bandit 9

2.7 Numerosity Comparison 10

3 Behavioral summary statistics **11**

4 Posterior predictive checks **12**

5 Intraclass correlations values computed based on all participants **12**

6 The implications of using a fully hierarchical model **12**

7 Behavioral task performance **13**

8 Excluded participants in the Go/No-Go and Lottery ticket tasks **13**

9 Change in the computational phenotype over time **13**

10 Model convergence diagnostics **13**

11 Parameter identifiability **14**

12 Go/No-Go: The consequences of model misspecification **14**

13 Intertemporal Choice: The effects of the computational model on parameter stability **15**

14 Daily survey questions **16**

15 Supplementary Figures **24**

1 Experimental tasks

To begin a task, participants first had to accept a prompt that expanded their browser window to full screen mode. They then received instructions about the general procedure, and how to perform each task. Two multiple-choice comprehension questions reflecting important instructions had to be correctly answered to proceed. If one or more of the questions was incorrectly answered, participants were redirected to the beginning of the instructions. Every task except for the Intertemporal choice and Lottery ticket tasks included a short practice block, which was a shortened version of the tasks. Each practice block had built in "suspicion checks". If a participant always chose the same answer, chose no answers, or had an unusually high rate of error, "suspicion checks" were triggered and participants were sent back to the beginning of the instructions. This could happen a maximum of three times. To incentivise participants to perform all tasks as instructed, monetary bonus, proportional to their performance level, was awarded. No performance-based bonus was earned in the Intertemporal Choice task and during practice blocks of all tasks.

At the end of each task, participants were asked if they experienced any technological issues or major distractions, and how engaged they were in the task. Participants were also given the opportunity to provide any other feedback they felt appropriate.

1.1 Go/No-Go

In the Go/No-Go task, participants were instructed on the four types of stimulus presented in each block: "Go to win", "No-Go to win", "Go to avoid punishment", and "No-Go to avoid punishment". Each of these rules was randomly shuffled to be represented by a pseudo-fractal image. After a 48 trial long practice block, participants completed three 80 trial long experimental blocks (20 trials per condition). Different images were associated with different conditions in each block, but the same images were reused each week. The rule attached to each image was determined anew every week.

Each trial began with an intertrial-interval (ITI) displaying a fixation cross centered on a blank screen for a duration of 750ms, 900ms, 1050ms, 1200ms, 1350ms, or 1500ms. Each ITI duration occurred the same number of times in a block, and were shuffled randomly between trials. The stimulus image was then displayed for up to 1000ms. If the participant chose the "Go" response by pressing the space bar, that ended the trial early. A "No-Go" response was implemented by waiting for 1500 ms until the stimulus disappeared. Stimulus images were immediately followed by 1000ms of feedback.

Feedback could either be positive, as indicated by a green thumbs up, negative as indicated by a red thumbs down, or neutral as indicated by a grey flat hand with the palm down. If a participant correctly chose the response in a "to win" rule stimulus, there was an 80% chance they would receive positive feedback and a 20% chance they would receive neutral feedback. If they incorrectly responded in a "to win" rule stimulus, there was a 80% chance they would receive neutral feedback and a 20% chance they would receive positive feedback. If a participant correctly responded to a stimulus whose rule is "to avoid punishment" there was an 80% chance they would receive neutral feedback, and a 20% chance they would receive negative feedback. If they incorrectly responded to the "to avoid punishment" stimulus, there was an 80% chance they would receive negative feedback, and a 20% chance they would receive neutral feedback.

1.2 Change Detection

In the Change Detection task participants saw a pair of sequentially presented images and were asked to determine if the images were the same (no-change type) or if they were different (change type), and how confident they were in that judgement [1]. Each image showed a number of colored squares arranged on an invisible ring with a dark grey background. Squares, by their HTML color name and RGB values could be: 'Yellow' (255, 255, 0), 'Lime' (0, 255, 0), 'Aqua' (0, 255, 255), 'Blue' (0, 0, 255), 'DarkOrchid' (153, 50, 204), 'Red' (255, 0, 0), 'Orange' (255, 165, 0), or 'LightPink' (255, 182, 193). These colors were chosen to be easily distinguishable from one another. Colors were not repeated in a given image when there were less than 8

squares being shown. In conditions that showed eight squares a color could be repeated no more than once and neither image in a pair could show two of the same colors next to one another.

Participants completed five experimental blocks, each 40 trials long and one practice block 14 trials long. In four of the five blocks, trials were divided evenly between change type and no-change type. During a change type trial only one square (the target) would change color. These blocks differed by the number of squares, three, four, six, or eight, shown in each image. The number of squares did not vary within the block. In the remaining experimental block, eight squares were shown, but the number of targets varied between one and four. Trials in this block were evenly divided between no change, one target changed, two targets changed, three targets changed, and four targets changed. Trial types were were shuffled within blocks. The order of blocks was randomly shuffled at the beginning of each experiment. The practice block was completed prior to the experimental blocks, and it included one of every trial and condition type with regard to change/no-change, number of squares shown, and number of targets changed.

Participants had 100ms to look at each image, and had a between-image gap of 1500ms. During this gap the screen remained dark grey and a white fixation cross was displayed in the center of the screen. After the second image a white screen appeared asking them if the images had been the same or different and participants indicated their choice with a mouse cursor. There was no time limit set for this question, and participants were instructed that accuracy was more important than speed in this task. Immediately following this question was another asking them to rank their confidence for the previous judgment. Participants had four buttons on their screen that allowed them to select with a mouse "Very Unconfident", "Somewhat Unconfident", "Somewhat Confident", or "Very Confident". After the two questions participants received 500ms of feedback that displayed, "Correct" in green text or "Incorrect" in red text.

1.3 Random Dot Motion

The Random Dot Motion task was taken from a standard random dot kinetogram task template on jsPsych [2]. Participants completed four experimental blocks, each containing 96 trials [3]. Experimental blocks were preceded by a practice block, 16 trials long. On every trial, participants were shown a cluster of 200 randomly distributed white dots on a dark grey background. Dots were confined to an invisible ellipse shaped boundary in the center of the screen. A percentage of the dots: 5%, 10%, 35%, or 50% moved in a coherent direction, either left of right. The remainder of the dots moved randomly within the boundary. Each direction and percentage of coherently moving dots occurred with equal frequency and were shuffled randomly within the blocks.

Participants had 1500ms to determine the direction of motion of the coherently moving dots. They indicated their choice by pressing the corresponding left or right arrow key on their keyboard. Trials ended when a decision was made. During the practice block, each trial was followed by a 300ms period of feedback. Feedback was displayed as colored text on the dark grey background and could be a red "Incorrect", green "Correct", yellow "Too Slow" (if they did not answer within the 1500 ms timeframe), or yellow "Too Fast" (if they answered in less than 250 ms). During the experimental (non-practice) blocks, no feedback was given if participant answered correctly, however, they did receive 300ms of feedback for being incorrect, too fast, or too slow. After the trial and/or feedback, a 300ms intertrial-interval showing a centered white fixation cross on a dark grey background was shown [4].

1.4 Lottery Ticket

In the Lottery Ticket task participants chose between a "risky" or a "safe" ticket to gamble on. The task had 3 blocks, which tested small, medium, or large amounts of monetary reward (see below). Each block was 10 trials long such that probabilities associated with any given monetary outcome spanned between 0% and 100% in increments of 10, and the sum of probabilities on each ticket totaled 100%. The probabilities were the same between tickets in a given trial. Participants used the right and left arrow keys on their keyboard to indicate their preference between one of the two tickets. Participants could make a response only starting

1500ms following trial onset to avoid random decisions.

The expected value for all tickets were calculated by first establishing the lower monetary reward offered in the risky ticket (henceforth referred to as the “risky lesser amount”). A Box-Muller function was used to determine this amount to be between $0.01-0.20 (1 cent increments) for the small bets block, between $1-5 ($0.25 increments) for the medium bets block, and between $5-15 ($0.50 increments) for the large bets block. The stimuli used for the task were generated anew each week by randomly sampling values from each block interval. The higher monetary reward on the risky ticket was the risky lesser amount divided by 0.026. The higher monetary reward on the moderate ticket was the risky lesser amount divided by 0.05. The lower monetary reward on the moderate ticket was the risky lesser amount divided by 0.0625. These amounts were rounded to the nearest increment corresponding to their block. The task consisted of three blocks, each 10 trials long. The order of the blocks was randomly shuffled at the start of each task.

It should be noted that for each block, there was one trial in which the “risky” ticket was not the riskier bet, as there was a 100% chance that the higher amount offered would be rewarded. This was included as a way of seeing if the participant was paying attention and these trials were not used for modeling purposes.

1.5 Intertemporal Choice

It was previously shown that people differ in assigning value to future consequences of their actions and in the impulsiveness of their decisions [5, 6]. In this task, participants used the right and left arrow keys on their keyboard to indicate their preference between a smaller amount of money available immediately or a larger amount of money available at a later time. Participants could make a response only starting 1000ms following trial onset to avoid random decisions.

Unlike the task used by Kirby [7], we did not use the exact same monetary rewards, delay times and order throughout the study. The specific variables in our version of the task were determined anew with each run of the task to minimize the risk of participants merely memorizing and repeating the same answers from week to week. Variables generated for each trial were consistent with other Intertemporal Choice studies. We used the same discount rate parameters (k): 0.00016, 0.00040, 0.0010, 0.0025, 0.0060, 0.016, 0.041, 0.10, and 0.25. as well as the same small ($25, $30, $35), medium ($50, $55, $60), and large ($75, $80, $85) delayed reward amounts (A) used in [7]. The delay (D) was bounded between 7 and 273 days. To determine the delay used and the amount of reward immediately available (V) the following hyperbolic function was implemented across the range of delays:

$$V = \frac{A}{1 + kD} \tag{1}$$

The amount of delay was chosen such that the amount of reward immediately available would be a whole number [8]. We used the hyperbolic function to determine the immediately available amount, such that with each given discount rate, the immediate amount and the discounted delayed account will be of equal size. Each group of delay reward (small, medium, large) was used three times in the weekly administration of the task, for a total of 27 trials (1 block). No performance-based bonus was given for this task.

1.6 Two-armed Bandit

In this task, participants were instructed to “play” one of two “slot machines” with the objective of maximizing their reward. Slot machines were represented by two side-by-side brightly colored rectangles the screen with the text “Risky” or “Safe” written in clear black font. The color of the rectangles remained constant within each game but changed between games. Participants played 30 sets of independent machines (games), each for 10 trials. It was emphasized in the instructions that the color of the machine had no bearing on the reward/loss and that, even if a color repeated in the task, there was no connection between the machines in different games. Each set of machines did not necessarily have one risky machine and one safe machine, a game could also be between two safe machines, or two risky machines. The designation for each machine

was chosen randomly prior to each game. The exception to this was the practice trial, which always had a safe machine vs risky machine.

Participants played a slot machine by clicking on one of the colored rectangles with their mouse cursor. After each decision feedback was displayed for 1000ms. Feedback had two lines of text: an upper line displayed how many coins were gained or lost and the font was either green or red based respectively; the lower line of text displayed in black their current coin balance.

Participants were given a starting "balance" of 250 coins. Every decision they made led to a gain or loss of coins. Instruction comprehension questions checked that they understood that "Risky" machines would lead to a different outcome every time and "Safe" machines would lead to the same outcome every time. The balance carried over between games. For each machine in a game the mean outcome was drawn from a normal distribution with a mean of 0 and a standard deviation of 100. The safe machine returned the same $\mu(S)$ outcome every time. The risky machine returned an outcome generated from $\mu(R)$ with a standard deviation of 16. Participants were informed that one machine was always better than its counterpart.

1.7 Numerosity Comparison

In this task, participants used the right and left arrow keys on their keyboard to indicate the "treasure chest" they believed had more dots ("gold coins") hovering over it. Once participants made a choice a rectangular white border briefly showed up around the chosen coins. This was followed by an ITI, which displayed a centered white fixation cross on a black background for 500 ms before the next stimulus.

The gold coins were filled bright yellow circles hovering mid-screen above an image of a treasure chest. The screen background was black and the treasure chest was brown. The number of coins in each trial was drawn from a distribution with a mean of 24 and standard deviation of 5. No two chests had the same amount of coins and coins did not touch or overlap. Half of the trials controlled for surface area, while the other half of the trials controlled for coin radius. In the trials that controlled for radius, the coins for both chests remained at a fixed size of 10 points and the number of coins was drawn from the distribution described above. In the trials that controlled for surface area, one chest (left or right) was randomly selected and displayed coins sized as the radii-controlled stimulus. The other chest had coins with either a larger or smaller radius such that the surface area for both chests was the same. The radii-controlled and surface-controlled trials were randomly shuffled within the task blocks.

First, participants completed a practice block, 16 trials long prior to two experimental blocks, each 80 trials long. During the practice trial, participants received feedback - a green "correct", red "incorrect", or grey "invalid response" screens that appeared for 500ms after their choice. Participants were made aware that they must answer within 1000ms or they receive an "invalid response" error. To move on to the experimental blocks from the practice block, participants had to have an error rate below 60%. If participants had an error rate above 60%, consistently chose only the right side or only the left side, or did not answer any of the trials, they were sent back to the instruction block. This could occur up to three times to ensure participants understand the task. Feedback was not given during the experimental blocks.

2 Computational models

2.1 Go/No-Go

We modeled participants' choices using a reinforcement learning model adapted from Guitart-Masip et al. [9]. The probability of choosing the action a on trial t, given the stimulus s, was calculated based on an action weight $\mathbf{W}_{(a_t,s_t)}$ which was passed through a softmax function:

$$P(a_t, s_t) = \left[\frac{\exp(W(a_t, s_t))}{\sum_{a'} \exp(W(a', s_t))}\right](1-\xi) + \frac{\xi}{2} \tag{2}$$

$$W(a_t, s_t) = \begin{cases} Q_t(a_t, s_t) + b + \pi V_t(s_t), & \text{if } a_t = \text{go} \\ Q_t(a_t, s_t), & \text{otherwise} \end{cases} \tag{3}$$

$$V_t(s_t) = V_{t-1}(s_t) + \epsilon(\rho r_t - V_{t-1}(s_t)) \tag{4}$$

$$Q_t(a_t, s_t) = Q_{t-1}(a_t, s_t) + \epsilon(\rho r_t - Q_{t-1}(a_t, s_t)) \tag{5}$$

with the following free parameters:

- ξ is the lapse rate ($0 < \xi < 1$)
- $\rho_{\text{rew}\backslash\text{pun}}$ is the effective size of reward/punishment reinforcement ($0 < \rho_{\text{rew}\backslash\text{pun}} < \infty$)
- ρ_{neut} is the effective size of neutral outcomes ($0 < \rho_{\text{neut}} < \infty$)
- b is the Go bias ($-\infty < b < \infty$)
- π is the Pavlovian bias ($-\infty < \pi < \infty$)
- ϵ is the learning rate ($0 < \epsilon < 1$)

Reinforcement on trial t was $r_t \in (-1, 1)$. The original model of Guitart-Masip et al. included only a single "effective size" parameter ρ, and neutral outcomes were modeled as $r_t = 0$. This reflects the assumption that neutral outcomes do not reinforce behavior, but simply allow the estimated values of $V_t(s_t)$ or $Q_t(a_t, s_t)$ to decay back to 0. Here, we modified this model in a simple but important way, by introducing an additional parameter of effective size for neutral outcomes. We further modeled neutral outcomes not as 0, but as ± 1 based on their context: -1 in reward conditions and $+1$ in punishment conditions. This reflects the assumption that neutral outcomes can reinforce behavior and that participants valuate neutral outcomes relative to the alternative outcome [10, 11].

2.2 Change Detection

Following Wilken & Ma [1], we used the absolute difference model (MAD) based on the absolute difference of two normally distributed variables with SDs σ and a distance d:

$$s_d(x) = \frac{1}{\sigma\sqrt{2\pi}}\left(e^{\frac{-(x-d)^2}{2\sigma^2}} + e^{\frac{-(x+d)^2}{2\sigma^2}}\right). \tag{6}$$

We set the distance d to 0 for no-change trials and to 1 for change trials. Fixing d to 1 reflects the simplifying assumption that a change between any two colors is represented similarly by the participants.

Thus, given a certain detection threshold θ, participants detect a change with probability:

$$p(\text{change detected}) = \int_{\theta}^{\infty} \mathrm{d}x\, s_d(x) \tag{7}$$

$$= \frac{1}{2}\left(\text{Erfc}\left(\frac{\theta - d}{\sqrt{2}\sigma}\right) + \text{Erfc}\left(\frac{\theta + d}{\sqrt{2}\sigma}\right)\right) \tag{8}$$

With this, the probability to hit, h (change detected|change occurred) or to false alarm, f (change detected|no change occurred) is:

$$h \equiv P(x > \theta|\text{change occurred}) \equiv \int_{\theta}^{\infty} \mathrm{d}x\, s_d(x) \text{ for } d > 0 \tag{9}$$

$$f \equiv P(x > \theta|\text{no change occurred}) \equiv \int_{\theta}^{\infty} \mathrm{d}x\, s_0(x) \text{ for } d = 0 \tag{10}$$

where noise is calculated as $s_0(x)$.
Thus, participants detect or do not detect a change on trial t with probability:

$$P_t(\text{change detected}|\text{change occurred}) = 1 - (1-h)^{T_t}(1-f)^{N_t - T_t} \tag{11}$$

$$P_t(\text{change detected}|\text{no change ocurred}) = 1 - (1-f)^{N_t} \tag{12}$$

$$P_t(\text{no change detected}|\text{no change occurred}) = (1-f)^{N_t} \tag{13}$$

$$P_t(\text{no change detected}|\text{change occurred}) = (1-h)^{T_t}(1-f)^{N_t - T_t} \tag{14}$$

with the following variables:

- T_t is number of changed squares on trial t
- N_t is number of presented squares on trial t

and the following free parameters:

- θ is the detection threshold ($0 < \theta < \infty$)
- σ is the noise level ($0 < \sigma < \infty$)

In general, θ and σ depend on the set size, i.e., the number of presented squares. Here we follow the results of [1] regarding the monotonic increase in σ and assume that both parameters scale linearly with set size:

$$\theta_t = \theta + s_\theta(N_t - N_{min}), \tag{15}$$

$$\sigma_t = \sigma + s_\sigma(N_t - N_{min}) \tag{16}$$

where θ_t and σ_t are the detection threshold and noise level in trial t, $s_\theta > 0$ and $s_\sigma > 0$ are the scaling factors, and N_{min} is the minimal number of squares presented in the experiment, in our case it was 3. With this formulation, the main parameters of interest in this task's phenotype are θ and σ.

2.3 Random Dot Motion

Response times (RT) on correct and incorrect trials were modeled using the drift diffusion model [12]:

$$\mathrm{RT}_{\mathrm{correct},t} \sim \mathrm{WFPT}(\alpha, \tau, 0.5, \delta c_t) \tag{17}$$

$$\mathrm{RT}_{\mathrm{incorrect},t} \sim \mathrm{WFPT}(\alpha, \tau, 0.5, -\delta c_t), \tag{18}$$

where WFPT is the Wiener first passage time distribution with the following free parameters:

- α is the boundary separation ($0 < \alpha < \infty$)
- τ is the non-decision time ($0 < \tau < \min(\mathrm{RT})$)
- δ is the drift rate ($0 < \delta < \infty$)

Note that we fixed the bias parameter to be 0.5 (no bias towards either of the choice options). In this task, the coherence level was $c_t \in (0.05, 0.1, 0.35, 0.5)$.

2.4 Lottery Ticket

Participants' choices were modeled with the utility and softmax functions [13, 14], where the probability to choose the *safe* option on trial t was:

$$P(a_t = \mathrm{safe}) = \frac{\exp(\beta v_{t,\mathrm{safe}})}{\exp(\beta v_{t,\mathrm{safe}}) + \exp(\beta v_{t,\mathrm{risky}})} = \frac{1}{1 + \exp(-\beta(v_{t,\mathrm{safe}} - v_{t,\mathrm{risky}}))} \tag{19}$$

with the following variables (showing for the *risky* ticket only):

- $v_{t,\mathrm{risky}} = \frac{U_{t,\mathrm{risky}}}{\sqrt{\mathrm{var}(U_{t,\mathrm{safe}}) + \mathrm{var}(U_{t,\mathrm{risky}})}}$
- $U_{t,\mathrm{risky}} = P_{t,\mathrm{high}} A^{\rho}_{t,\mathrm{high}} + P_{t,\mathrm{low}} A^{\rho}_{t,\mathrm{low}}$
- $U_{t,\mathrm{risky}}$ is the utility of a ticket with the wider monetary value range
- $v_{t,\mathrm{risky}}$ is the standardized utility of a ticket with the wider monetary value range
- $A_{t,\mathrm{high}}$ is the higher monetary value in trial t
- $A_{t,\mathrm{low}}$ is the lower monetary value in trial t
- $P_{t,\mathrm{high}}$ is the probability of winning the higher monetary value in trial t
- $P_{t,\mathrm{low}}$ is the probability of winning the lower monetary value in trial t

and the following free parameters:

- ρ is the risk attitude ($0 < \rho < \infty$)
- β is the inverse temperature ($0 < \beta < \infty$)

The square root term serves as a standardization introduced in [14] as a means to reduce the correlation between the two model parameters and promote parameter identifiability.

2.5 Intertemporal Choice

Discount curves and participants' choices were modeled by the hyperbolic and softmax functions [8], where the probability to choose the *later* option on trial t was:

$$P(a_t = \mathrm{later}) = \frac{\exp(\beta V_{t,\mathrm{later}})}{\exp(\beta V_{t,\mathrm{later}}) + \exp(\beta V_{t,\mathrm{sooner}})} = \frac{1}{1 + \exp(-\beta(V_{t,\mathrm{later}} - V_{t,\mathrm{sooner}}))} \tag{20}$$

$$V_t = \frac{A_t}{1 + kD_t} \tag{21}$$

with the following variables:

- V_t is the discounted value on trial t
- D_t is the delay time in days (for *sooner* options, D_t=0)
- A_t is the non-discounted value

and the following free parameters:

- k is the discount rate ($0 < k < \infty$)
- β is the inverse temperature ($0 < \beta < \infty$)

In addition, we explored the effect of using the orignal model proposed by [7], which does not account for choice stochasticity, and includes only the discount rate parameter k. This model cannot easily be fit using a Bayesian approach since no stochasticity is introduced into the decision rule. Therefore, we fit the discount rate independently for each sessions, as done in [7]: For each trial, we calculated the discount rate that predicts an indifferent choice between the two amounts (indifference k). We ordered the indifference k's by magnitude and calculated the geomteric mean of every two consecutive values. Together with the minimal and maximal indifference k's, these values comprise all possible discount rates k that a participant can be assigned in a particular session. Since a single k value might not be consistent with all the choices made by a participant, we chose the k value that yielded the greatest proportion of consistent choices.

2.6 Two-Armed Bandit

We modeled participants' choices using probit regression. Following [15], for every trial t we calculated the following values:

- Estimated value difference $(V) : Q_t(1) - Q_t(2)$
- Relative uncertainty $(RU) : \sigma_t(1) - \sigma_t(2)$
- Total uncertainty $(TU) : \sqrt{\sigma_t^2(1) + \sigma_t^2(2)}$

where $Q_t(k)$ and $\sigma_t(k)$ are the mean and standard deviation of rewards from machine k on trial t. We entered normalized V, RU and $\text{sign}(V)/TU$ as regressors into the probit regression model and fitted their regression coefficients ($-\infty < \mathbf{W} < \infty$). We normalized the regressors by dividing their raw valued by the standard deviation of values across all trials and sessions, to ensure that all regressors are scaled similarly. Choice probability on trial t was modeled as:

$$P_{(a_t=1|\mathbf{W})} = \Phi\left(w_1 V + w_2 RU + w_3 \frac{\text{sign}(V)}{TU}\right) \tag{22}$$

where $\Phi(\cdot)$ is the cumulative distribution function of the standard normal distribution. Notice that we used $\text{sign}(V)/TU$ rather than V/TU as was done in the original paper. Without introducing this modification, the model would not converge, likely due to the collinearity between the predictors for V and V/TU. We further verified that this modified model was able to fit the data in [15], and in fact improved the model fit (BIC improved from 12,689 to 12,560; AIC improved from 12,621 to 12,492).

We used Kalman filter to recursively compute the posterior mean $Q_t(k)$ and variance $\sigma_t^2(k)$ of the chosen machine k ($k \in 1, 2$) on trial t:

$$Q_{t+1}(a_t) = Q_t(a_t) + \alpha_t(r_t - Q_t(a_t)) \tag{23}$$

$$\sigma_{t+1}^2(a_t) = \sigma_t^2(a_t) - \alpha_t \sigma_t^2(a_t) \tag{24}$$

where the learning rate α_t is given by:

$$\alpha_t = \frac{\sigma_t^2(a_t)}{\sigma_t^2(a_t) + \tau^2(a_t)}, \tag{25}$$

where $\tau^2(a_t)$ is the true reward variance for the chosen machine at trial t (36 for the Risky machine; 0.00001 for the Safe machine, to avoid numerical issues of setting the variance to 0).

2.7 Numerosity Comparison

Due to noise, participants encode the number on circles on the left (a) and the number of circles on the right (b) as $\hat{a}$ and $\hat{b}$, where:

$$\hat{a} \sim \mathcal{N}(a, w^2 a^2) \tag{26}$$

$$\hat{b} \sim \mathcal{N}(b, w^2 b^2) \tag{27}$$

with the free parameter w $(0 < w < \infty)$ is the Weber fraction [16]. Participants respond "left" if $\hat{a} > \hat{b}$, where:

$$\hat{a} - \hat{b} \sim \mathcal{N}(a - b, w^2(a^2 + b^2)) \tag{28}$$

Therefore, for every (a,b), the probability of choosing "left" is calculated as follows:

$$p(a_t = \text{left}) = \int_{-\infty}^{0} \mathcal{N}(x;\, a - b, w^2(a^2 + b^2))dx \tag{29}$$

3 Behavioral summary statistics

Table S1: Behavioral measures of accuracy and reaction times across task conditions (mean ± standard deviation).

Task	Condition	Accuracy	RT (s)
Go/No-Go	Go2Win	0.85±0.12	0.54±0.05
	No-Go2Win	0.62±0.28	0.58±0.05
	Go2Avoid	0.64±0.29	0.57±0.06
	No-Go2Avoid	0.84±0.1	0.60±0.06
Change detection	No target	0.83±0.08	1.23±0.69
	One target	0.68±0.08	1.16±0.32
	Set size of 8	0.70±0.06	1.29±0.81
Random dot motion	5% coherence	0.61±0.03	0.84±0.15
	10% coherence	0.73±0.06	0.81±0.15
	35% coherence	0.96±0.05	0.66±0.13
	50% coherence	0.98±0.04	0.63±0.13
Lottery ticket	Small difference	-	2.91±0.67
	Medium difference	-	2.77±0.74
	Large difference	-	2.71±0.72
Intertemporal choice	Short delay	-	2.02±0.49
	Medium delay	-	2.09±0.64
	Long delay	-	2.03±0.58
Two-armed bandit	Risky-Risky	0.75±0.03	0.44±0.17
	Safe-Safe	0.89±0.02	0.41±0.14
	Risky-Safe/S-R	0.79±0.04	0.44±0.16
Numerosity comparison	-	0.85±0.04	0.62±0.07

4 Posterior predictive checks

To verify that the model parameters capture participants' behavior in all seven tasks, we calculated posterior predictive checks. The estimated participants' behavior based on the computational phenotype derived from the dynamic model and the actual behavior is presented in Supplementary Figure S8.

5 Intraclass correlations values computed based on all participants

Figure 2 in the main text shows the ICC values across the phenotype. Since ICC cannot be calculated in the presence of missing data, there we used only participants that had no missing sessions. An alternative is to use data from all participants, but include only the maximal number of sessions in each task, for which all participants have data. Supplementary Figure S1 shows the stability of the computational phenotype using this alternative calculation. The results are qualitatively similar to those presented in the main text.

6 The implications of using a fully hierarchical model

Figure 2 in the main text shows the ICC values based on the independent hierarchical model we used to fit the data in the main text. This model includes two levels of hierarchy, where participant-specific priors are sampled from a population-level distribution, and session-specific phenotype parameters are sampled around the participant-specific prior. To test whether accounting for the hierarchical strucutre of the data promotes phenotype stability, we repeated the analysis with the reduced statistical model. In this model, all sessions across all participants are considered as independent, such that the phenotype parameters are sampled around a population-level value (as opposed to the indepndent model, where the phenotype parameters are sampled from session-specific and participant-specific distributions). Supplementary Figure S4 shows the ICC values for the reduced model. Compared with Figure 2 in the main text, it is clear that the ICC values are much lower for all phenotype parameters estimated using the reduced model. This result is in line with previous work that found higher test-retest reliability with hierarchical model fitting, compared with methods like maximum likelihood estimation [17, 18, 19].

The advantage of using a fully hierarchical model for parameter estimation is even more striking in the case of simulated data. As described in the main text, when calculating an upper bound for the ICC values, we simulated data based on a known phenotype, which was fixed in time. An ideal parameter estimation method would yield ICC values of 1 for all parameters, since the ground truth phenotype is fixed in time. By pooling information across participants and sessions, the hierarchical model yielded highly stable parameter estimates, resulting in ICC values very close to 1 (red vertical lines in Figure 2 in the main text). In contrast, using the same simulated dataset as input, the reduced model yielded much lower ICC values. Supplementary Figure S3 is identical to Figure 2 in the main text, but includes also the estimated upper bounds of ICC based on the reduced model (marked with triangles).

For completeness, in Supplementary Figure S2 we provide a visualization of the variance components used to calculate the ICC: between-participant variance, between-session variance and error variance. The results for the independent model are shown on the left and the results for the reduced model are shown on the right. The reduced model doesn't account for the full hierarchical structure of the data (all phenotype parameters were sampled from a population-level distribution, as opposed to a session level nested within a participant level). Notably, in this calculation the error variance is generally much larger than the between-session variance. This is true even for phenotype parameters for which the analysis in the main text identified a greater contribution of (between-session) practice effects compared with noise, such as the effective size of neutral outcomes (ρ_{neut}) in the Go/No-Go task. This highlights a limitation in the interpretation of between-session variance in the ICC calculation: This source of variance only accounts for systematic changes between sessions across participants [20, 21]. In other words, if practice effects are participant-specific, they will be

absorbed into the error variance. The same is true for effects of participant-specific affect. By explicitly modeling different sources of variance, the approach we present in Figure 4 in the main text allowed us to more properly depict the relative contribution of known sources (practice and affect) and unknown sources (noise) to the phenotype dynamics.

7 Behavioral task performance

For examining how behavioral task performance changed throughout the study, we calculated the mean accuracy for each week and in each task with objectively defined performance criteria. As can be seen from Supplementary Figure S6, perforamnce in nearly all tasks increased during the 12-week long repetitive testing.

8 Excluded participants in the Go/No-Go and Lottery ticket tasks

To promote convergence of the Markov chain Monte Carlo procedure for parameter estimation, we excluded participants that showed atypical behavior in the Go/No-Go and Lottery Ticket tasks. In the Go/No-Go task, we excluded participants defined as "non-learners", namely participants who had an average accuracy of less than 0.55 throughout the study, which resulted in excluding 24 participants (for comparison, [9] found a similar fraction of "non-learners", 11 out of 30 participants). Here we provide an analysis of these participants. Figures S12, S13, and S14 show the average task performance in "non-learners", their posterior predictive checks, and the phenotype dynamics, respectively (compare with "learners" shown in Figures 3 and 4 in the main text). It is clear from Supplementary Figure S13 that these participants fail to respond correctly to the two anti-Pavlovian conditions, "No-Go to win" and "Go to avoid punishment". This clear difference in behavior justifies the separate analysis of these two participant groups (as was done in [9]).

In the Lottery ticket task, we excluded participants who consistently chose one of the options (either "Safe" or "Risky" ticket) in over 80% of the trials (these were all participants who chose the "Safe" option). This resulted in the exclusion of 32 participants. Supplementary Figure S16 and S17 show the posterior predictive checks and the phenotype dynamics for these excluded participants, respectively (compare with Figure 5 in the main text and Supplementary Figure S8). Here, too, we found notable differences in the phenotype parameters of the two groups, with a difference of two orders of magnitude for both risk attitude ρ and inverse temperature β. Notably, and in agreement with participants' almost deterministic "Safe" choices, the risk attitude values are tightly distributed around an extremely low value, 2×10^{-3}.

9 Change in the computational phenotype over time

Similar to Figure 4 in the main text showcasing temporal variability in the Go/No-Go computational phenotype, we examined the temporal dynamics of the computational phenotype in the remaning 6 tasks (Supplementary Figure S7)

10 Model convergence diagnostics

To verify that the Markov chain Monte Carlo procedure for parameter estimation exhibited satisfactory convergence, we utilized the convergence summary statistic $\hat{R}$, as well as the number of divergent transitions encountered during sampling. We verified that all population-level parameters exhibited $\hat{R} < 1.05$ [22]. Since each task included thousands of parameters (given $\sim 12 \times 90 = 1080$ task sessions), we did allow for occasional participant-specific session-level parameters that exceeded this $\hat{R}$ threshold. In addition, for some tasks, posterior sampling of the dynamic model resulted in a small percentage of divergent transitions that we were not able to eliminate. Visualization did not indicate a specific part of the posterior distribution that suffered from these divergent transitions. Here, we report the incidences of high $\hat{R}$ and divergent transitions.

- Go/No-Go:
  Independent model - Three parameters had $\hat{R} > 1.05$ for session 9 of participant 4, a single parameter for session 3 of participant 61, and a single parameter for session 1 of participant 54.
  Dynamic model - Three parameters had $\hat{R} > 1.05$ for session nuber 3 of participant number 61.
- Lottery Ticket:
  Independent model - 2.1% divergent transitions.
  Dynamic model - 4% divergent transitions.

11 Parameter identifiability

To assess parameter identifiability, we used the phenotype estimates (mean of the posterior) from the independent hierarchical model to simulate data for each session and participant. This resulted in a simulated dataset with known parameters, whose distribution is similar to that of the empirical data. Then, we fit the independent model to these simulated data and compared the resulting phenotype to the ground-truth generating values. We used ICC as a quantitative measure of parameter identifiability, where the generating and recovered parameters can be thought of as test and retest measures. Overall, we found very good identifiability across all tasks, with only a few parameters with values of ICC$<$ 0.9:

- Intertemporal Choice: Discount factor - 0.99; Inverse temperature - 0.91.
- Lottery Ticket: Risk attitude - 0.92; Inverse temperature - 0.65.
- Go/No-Go: Pavlovian bias - 0.95; Learning rate - 0.9; Lapse rate - 0.93; "Go" bias - 0.92; Effective size of reward/punishment - 0.5; Effective size of neutral outcomes - 0.73.
- Change Detection: Sensitivity - 0.98; Detection threshold - 0.96.
- Random Dot Motion: Drift rate - 0.96; Non-decision time - 0.97; Boundary separation - 0.65.
- Two-Armed Bandit: Relative uncertainty - 0.95; Estimated value difference - 0.93; Total uncertainty - 0.8.
- Numerosity Comparison: Weber fraction - 0.92.

12 Go/No-Go: The consequences of model misspecification

As explained in the main text, the original Pavlovian RL model of Guitart-Masip et al. [9] did not capture participants' behavior in our dataset, particularly their responses in the punishment conditions (Supplementary Figure S9; importantly, previously suggsted modifications to the model, including separate learning rates or effective outcome size for reward/punishment also failed in fitting this data). Here, we highlight the consequences of fitting this misspecified model in terms of the phenotype dynamics.

Supplementary Figure S11 shows that using the original model of [9], we found a certainly existing effect of practice (pd=100) for the learning rate and the Pavlovian bias. This is in contrast to the modified model we propose, for which we did not find these effects, and instead found a prominent practice effect for the new parameter we introduced, the effective size of neutral outcomes. This demonstrates the consequences of model misspecification: Failure to include this additional parameter results in a misinterpretation of the underlying cognitive process. The variability that can be best explained by the missing parameter is now attributed to changes in the learning rate. While these changes allow the model to fit the first few trials in the punishment conditions (likely when participants get punished for making the wrong choice), they fail to capture their gradual improvement over the course of the session. This serves as a compelling example of the effects of model misspecification. Fortunately, in this case, the model misfit was clearly evident in the posterior predictive checks, signaling the need for the modification we made to the model.

Interestingly, both models perform similarly in fitting the first session (Supplementary Figure S10). However, as participants gained practice in later sessions, our modified model demonstrates an advantage in explaining the data. This is clearly depicted in Supplementary Figure S10, where the two models exhibit similar log-likelihoods for the first session, but a substantially better log-likelihood for the modified model in subsequent sessions.

13 Intertemporal Choice: The effects of the computational model on parameter stability

In the main text, we modeled the data from the Intertemporal choice task using a hyperbolic discount function with a stochastic softmax choice rule. Supplementary Figure S18 shows that this computational model (used in both the independent and reduced models) yields higher ICC values (median 0.93) compared with the original hyperbolic discount model proposed by [7], which does not allow for choice stochasticity (median ICC 0.68). In part, this can be attributed to the statistical model that is used for parameter estimation. As discussed in the main text, accounting for the hierarchical structure of the data promotes higher parameter stability. In line with this notion, the independent model yields the highest ICC values, followed by the reduced model that ignores that participant-level structure (reduced model median ICC 0.81). The lowest ICC values are obtained for the original hyperbolic discount model (median 0.68), for which the discount rate was estimated independently for each session without the softmax choice rule.

Practice effects persist after the first session

A potential concern regarding the practice effects we observed in our study is that they result from insufficient practice prior to the first session, such that participants become sufficiently familiar with the task only after completing the first session. However, the gradual increase in accuracy and in many of the computational parameters suggests that the practice effects we observed do persist over multiple sessions. To provide further support for this claim, we repeated the fitting of the dynamic hierarchical model to all tasks, this time excluding the first session. The probability of direction (pd) of the practice effects remained similar across the computational phenotype, with the exception of two parameters: in the Go/No-Go task, both the lapse rate ξ and the learning rate ϵ, which previously showed no clear practice effects, now showed negative practice effects (pd=97 and pd=100, respectively).

14 Daily survey questions

The following are the questions that were presented to the participants every session. Note that question 5 was presented with a typo, referring to the participant's momentary feelings "right now" rather than "in the past 24 hours".

1. How much did you feel nervous or anxious over the past 24 hours?

1) Very slightly or not at all

2) A little

3) Moderately

4) Quite a bit

5) Extremely

2. How much did you feel excited or enthusiastic over the past 24 hours?

1) Very slightly or not at all

2) A little

3) Moderately

4) Quite a bit

5) Extremely

3. How much did you feel upset over the past 24 hours?

1) Very slightly or not at all

2) A little

3) Moderately

4) Quite a bit

5) Extremely

4. How much did you feel irritable over the past 24 hours?

1) Very slightly or not at all

2) A little

3) Moderately

4) Quite a bit

5) Extremely

5. How much did you feel alert or attentive right now?

1) Very slightly or not at all

2) A little

3) Moderately

4) Quite a bit

5) Extremely

6. How much did you feel determined over the past 24 hours?

1) Very slightly or not at all

2) A little

3) Moderately

4) Quite a bit

5) Extremely

7. How much did you feel angry or hostile over the past 24 hours?

1) Very slightly or not at all

2) A little

3) Moderately

4) Quite a bit

5) Extremely

8. How much did you feel happy over the past 24 hours?

1) Very slightly or not at all

2) A little

3) Moderately

4) Quite a bit

5) Extremely

9. How much did you feel sad or down over the past 24 hours?

1) Very slightly or not at all

2) A little

3) Moderately

4) Quite a bit

5) Extremely

10. How much did you feel relaxed or at ease over the past 24 hours?

1) Very slightly or not at all

2) A little

3) Moderately

4) Quite a bit

5) Extremely

11. How much did you feel lonely over the past 24 hours?

1) Very slightly or not at all

2) A little

3) Moderately

4) Quite a bit

5) Extremely

12. How much did you feel outgoing over the past 24 hours?

1) Very slightly or not at all

2) A little

3) Moderately

4) Quite a bit

5) Extremely

13. How much did you feel dissatisfied with yourself over the past 24 hours?

1) Very slightly or not at all

2) A little

3) Moderately

4) Quite a bit

5) Extremely

14. How much did you feel stressed over the past 24 hours?

1) Very slightly or not at all

2) A little

3) Moderately

4) Quite a bit

5) Extremely

15. If you felt stressed out over the last 24 hours, how successfully were you at managing those stressful feelings?

0) N/A – I have not felt stressed out

1) Very unsuccessful

2) A little bit successful

3) Moderately successful

4) Very successful

5) Extremely successful

16. How much is your stress interfering with your day today?

1) Very slightly or not at all

2) A little

3) Moderately

4) Quite a bit

5) Extremely

17. How much did you feel stressed about social relationships (friends, roommate, family, partner, etc.) over the past 24 hours?

1) Very slightly or not at all

2) A little

3) Moderately

4) Quite a bit

5) Extremely

18. How much did you feel stressed about work and/or academics (deadlines, performance, etc.) over the past 24 hours?

1) Very slightly or not at all

2) A little

3) Moderately

4) Quite a bit

5) Extremely

19. How much did you feel stressed about other reasons (health, financials, extracurriculars, etc.) over the past 24 hours?

1) Very slightly or not at all

2) A little

3) Moderately

4) Quite a bit

5) Extremely

20. How much did you feel sick, ill, or in physical discomfort in the past 24 hours?

1) I felt unusually healthy

2) I felt normal

3) I felt a little under the weather or uncomfortable but went about my normal activities

4) I felt sick but tried to do a few important things

5) I felt too sick to do anything

21. Are you menstruating today?

0) Not Applicable

1) No

2) Uncertain - maybe just beginning

3) Yes with light flow

4) Yes with medium flow

5) Yes with heavy flow

22. How would you gauge your energy level today?

1) Little energy or motivation to do much of anything

2) Enough energy to get by but not enough to be very productive

3) Typical energy level with usual productivity

4) Plenty of energy to be even more productive than usual

5) Unusually high energy feeling hyper or even agitated at times

23. How much were you physically active during the past 24 hours?

1) Very slightly or not at all

2) A little

3) Moderately

4) Quite a bit

5) Extremely

24. How did you sleep last night?

1) Terribly: little or no sleep

2) Not so well: got some sleep but not enough

3) Sufficient: got enough sleep to function

4) Good: got a solid night's sleep and feel well-rested

5) Exceptional: one of my best nights of sleep

25. How many times did you check your phone last night during your intended sleep time?

1) Not at all

2) One time

3) Two times

4) Three to five times

5) More than five times

26. How much did you eat today?

1) Almost nothing at all

2) Less than I should have

3) Typical amount

4) I overate

5) Extreme overeating including food binge

27. How healthfully do you feel you ate today?

0) N/A - I did not eat today

1) Only unhealthy foods

2) Mostly unhealthy foods

3) Some healthy food and some unhealthy foods

4) Mostly healthy foods

5) Only healthy foods

28. In the past 24 hours, how much caffeine (e.g. coffee, tea, soft drink) did you consume?

1) None

2) One cup of coffee, tea, or soft drink (8oz)

3) Two cups of coffee, tea, or soft drink (16oz)

4) Three cups of coffee, tea, or soft drink (24oz)

5) Four or more cups of coffee, tea, or soft drink (32+oz)

29. In the past 24 hours, how much alcohol did you consume?

1) None

2) One alcoholic drink

3) Two alcoholic drinks

4) Three or four alcoholic drinks

5) Five or more alcoholic drinks

30. In the past 24 hours, how many cigarettes/cigars did you smoke?

1) None

2) Four or less

3) Five to ten

4) Eleven to twenty

5) Twenty-one or more

31. In the past 24 hours, when was the last time you used a vape pen (e-cigarette or similar)?

0) I have never used a vape and/or e-cigarette

1) In the last hour

2) Two to four hours ago

3) Four to twelve hours ago

4) Twelve to twenty-four hours ago

5) Two to five days ago

6) Five or more days ago

32. When was the last time you consumed any drugs (marijuana, recreational pills, etc.)?

0) I have never consumed drugs

1) In the last four hours

2) Four to twelve hours ago

3) Twelve to twenty four hours ago

4) One to two days ago

5) Two to five days ago

6) Five or more days ago

33. The last time you consumed drugs (marijuana, recreational pills, etc.), how impaired did you get?

0) N/A – I have never consumed drugs

1) Very slightly or not at all

2) A little

3) Moderately

4) Quite a bit

5) Extremely

34. Please describe anything else about your physical or mental state right now that would be useful to understand your health and feelings today, including listing any new or temporary medications you are taking.

Screen to write in was presented.

35. Did you engage in sexual activities with another person in the last 24 hours?

0) Prefer not to answer

1) No, and I feel negative about it (e.g. sad, bad, guilty, angry, etc.)

2) No, and I feel neutral about it (e.g. no strong feelings either way)

3) No, and I feel positive about it (e.g. happy, excited, glad, etc.)

4) Yes, and I feel negative about it (e.g. sad, bad, guilty, angry, etc.)

5) Yes, and I feel neutral about it (e.g. no strong feelings either way)

6) Yes, and I feel positive about it (e.g. happy, excited, glad, etc.)

36. Did you engage in sexual activities by yourself (e.g. masturbation) in the last 24 hours?

0) Prefer not to answer

1) No, and I feel negative about it (e.g. sad, bad, guilty, angry, etc.)

2) No, and I feel neutral about it (e.g. no strong feelings either way)

3) No, and I feel positive about it (e.g. happy, excited, glad, etc.)

4) Yes, and I feel negative about it (e.g. sad, bad, guilty, angry, etc.)

5) Yes, and I feel neutral about it (e.g. no strong feelings either way)

6) Yes, and I feel positive about it (e.g. happy, excited, glad, etc.)

15 Supplementary Figures

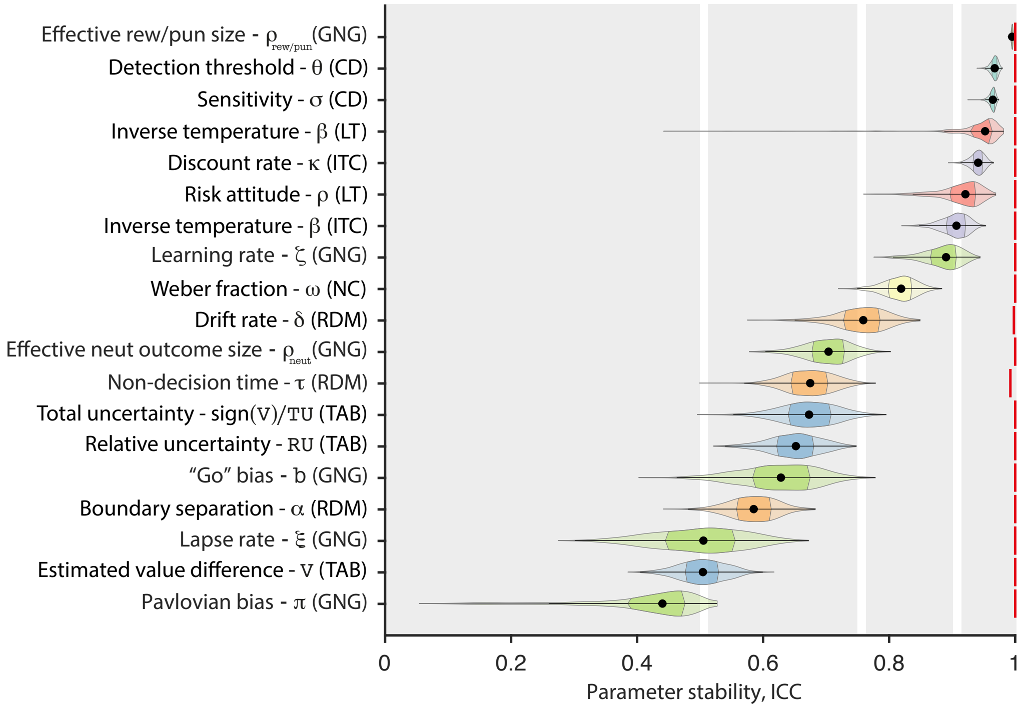

Figure S1: **Calculation of the stability of the computational phenotype based maximum sessions available for each participant**. Violin plots show the bootstrapped stability estimates in terms of intra-class correlations (ICC) for each parameter in the computational phenotype (1000 bootstrap iterations over participants). Points mark the median, shaded areas mark the inter-quartile range. Vertical red lines mark the upper stability bound estimated using simulated data. Gray regions mark a common interpretation of ICC values [23]: < 0.5 (poor), $0.5 - 0.75$ (moderate), $0.75 - 0.9$ (good), and > 0.9 (excellent). ICC was calculated only on the maximal number of sessions that existed for all participants: Go/No-Go - 9 sessions out of 12 (9/12; GNG), Change detection - 8/12 (CD), Intertemporal choice - 7/12 (ITC), Lottery ticket - 8/12 (LT), Numerosity comparison - 8/12 (NC), Two-armed bandit - 8/12 (TAB), Random-dot motion - 9/12 (RDM).

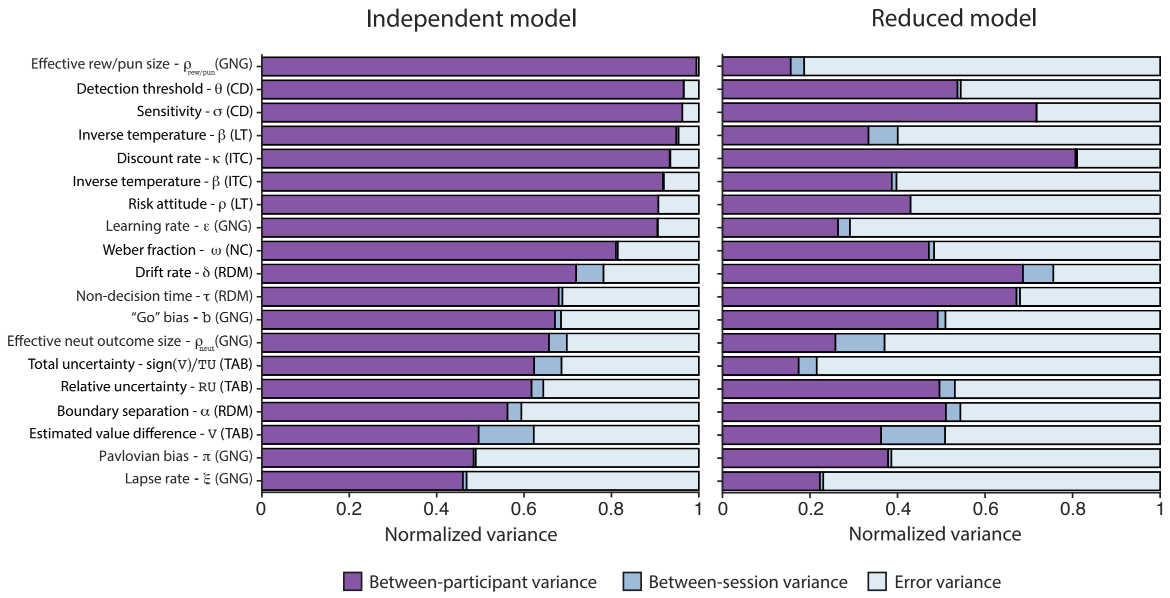

Figure S2: **Variance components of the ICC**. We estimated the contribution of each variance component for the stability measures of the computational phenotype parameters. To illustrate the effect of incorporating the full hierarchical structure in the independent model (left), we repeated the analysis for ICC values based on the reduced model (right), which does not account for the hierarchical structure of the data, namely a session-level nested within a participant-level.

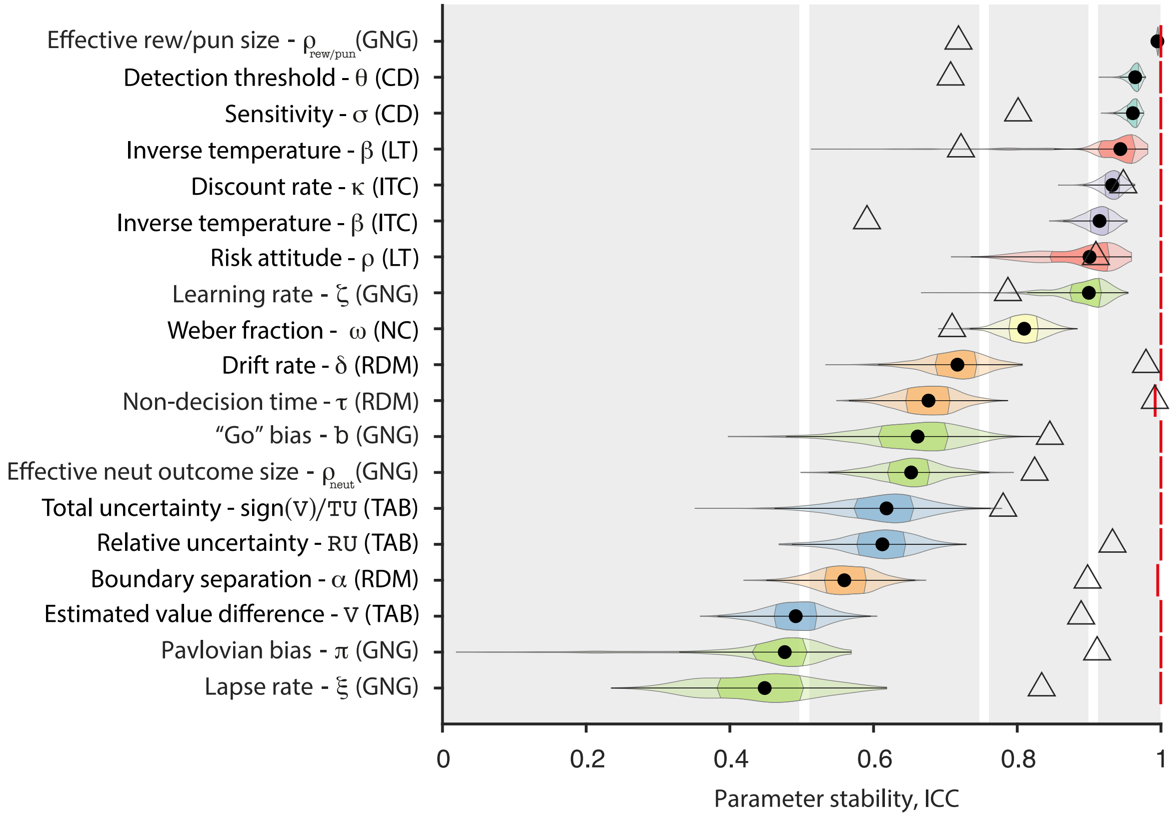

Figure S3: **Stability of the computational phenotype with upper bound**. Violin plots show the bootstrapped stability estimates in terms of intraclass correlations (ICC) for each parameter in the computational phenotype (1000 bootstrap iterations over participants; viloin colors indicate tasks) estimated using the independent model. Points mark the median, shaded areas mark the inter-quartile range. Vertical red lines mark the upper stability bound estimated using simulated data with a phenotype that is fixed in time. The triangles represent the upper ICC boundary estimated based on the reduced model, which does not exploit the full hierarchical structure of the data. Gray regions mark a common interpretation of ICC values [23]: < 0.5 (poor), $0.5 - 0.75$ (moderate), $0.75 - 0.9$ (good), and > 0.9 (excellent).

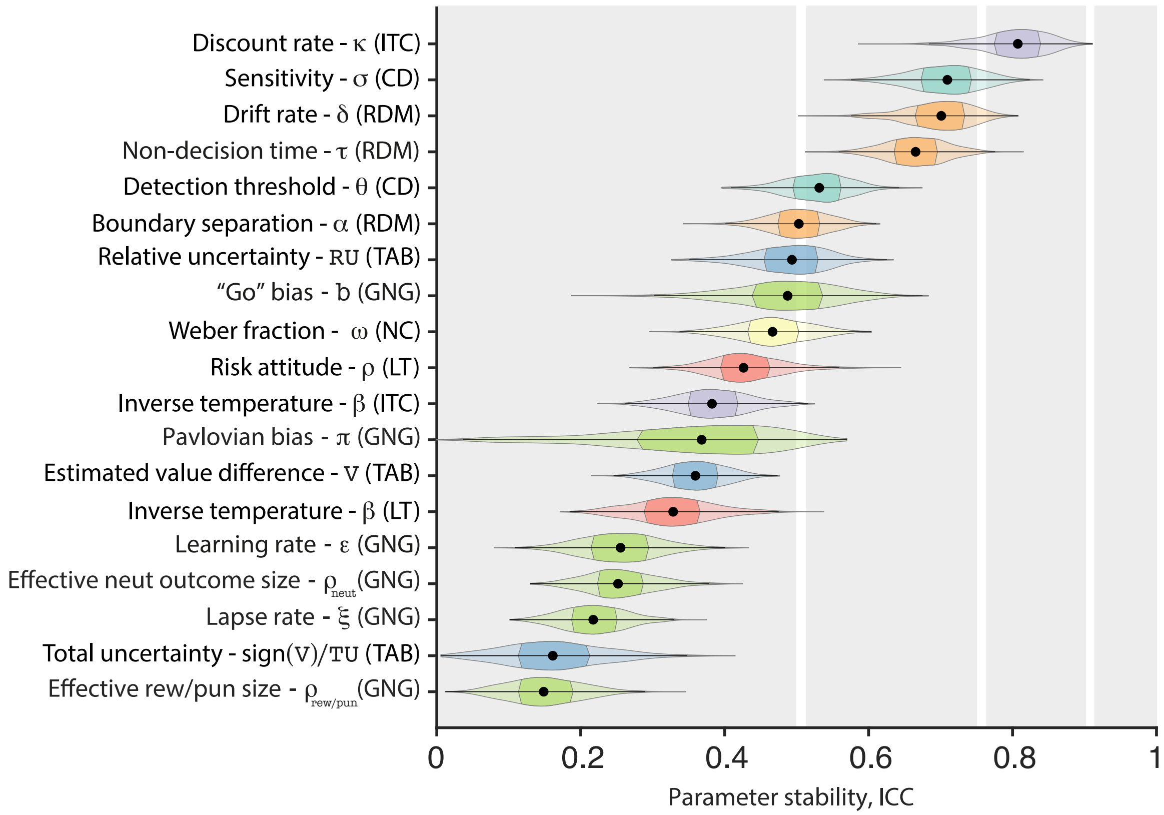

Figure S4: **Stability of the computational phenotype estimated using the reduced statistical model**. Violin plots show the bootstrapped stability estimates in terms of intraclass correlations (ICC) for each parameter in the computational phenotype (1000 bootstrap iterations over participants; viloin colors indicate tasks). Points mark the median, shaded areas mark the inter-quartile range. Gray regions mark a common interpretation of ICC values [23]: < 0.5 (poor), $0.5 - 0.75$ (moderate), $0.75 - 0.9$ (good), and > 0.9 (excellent). Compare to Figure 2 in the main text where we present the ICC values for the computational phenotype parameters estimated using the independent model.

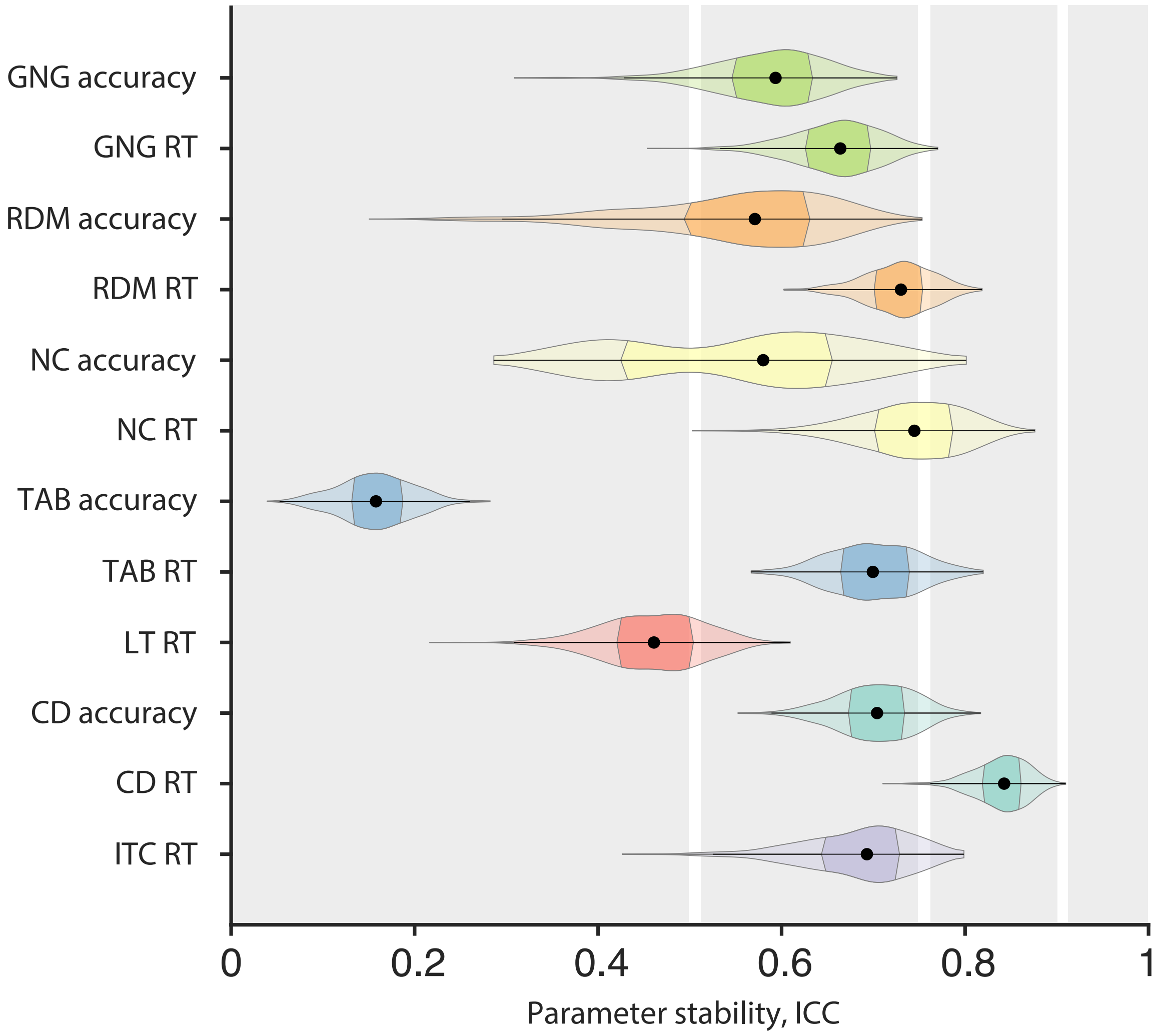

Figure S5: **Stability of the behavioral measures in each task**. Violin plots show the bootstrapped stability estimates in terms of intraclass correlations (ICC) for each behavioral measure in each task, either accuracy or response time (RT; 1000 bootstrap iterations over participants; viloin colors indicate tasks). Points mark the median, shaded areas mark the inter-quartile range. Vertical red lines mark the upper stability bound estimated using simulated data with a phenotype that is fixed in time. Gray regions mark a common interpretation of ICC values [23]: < 0.5 (poor), $0.5 - 0.75$ (moderate), $0.75 - 0.9$ (good), and > 0.9 (excellent).

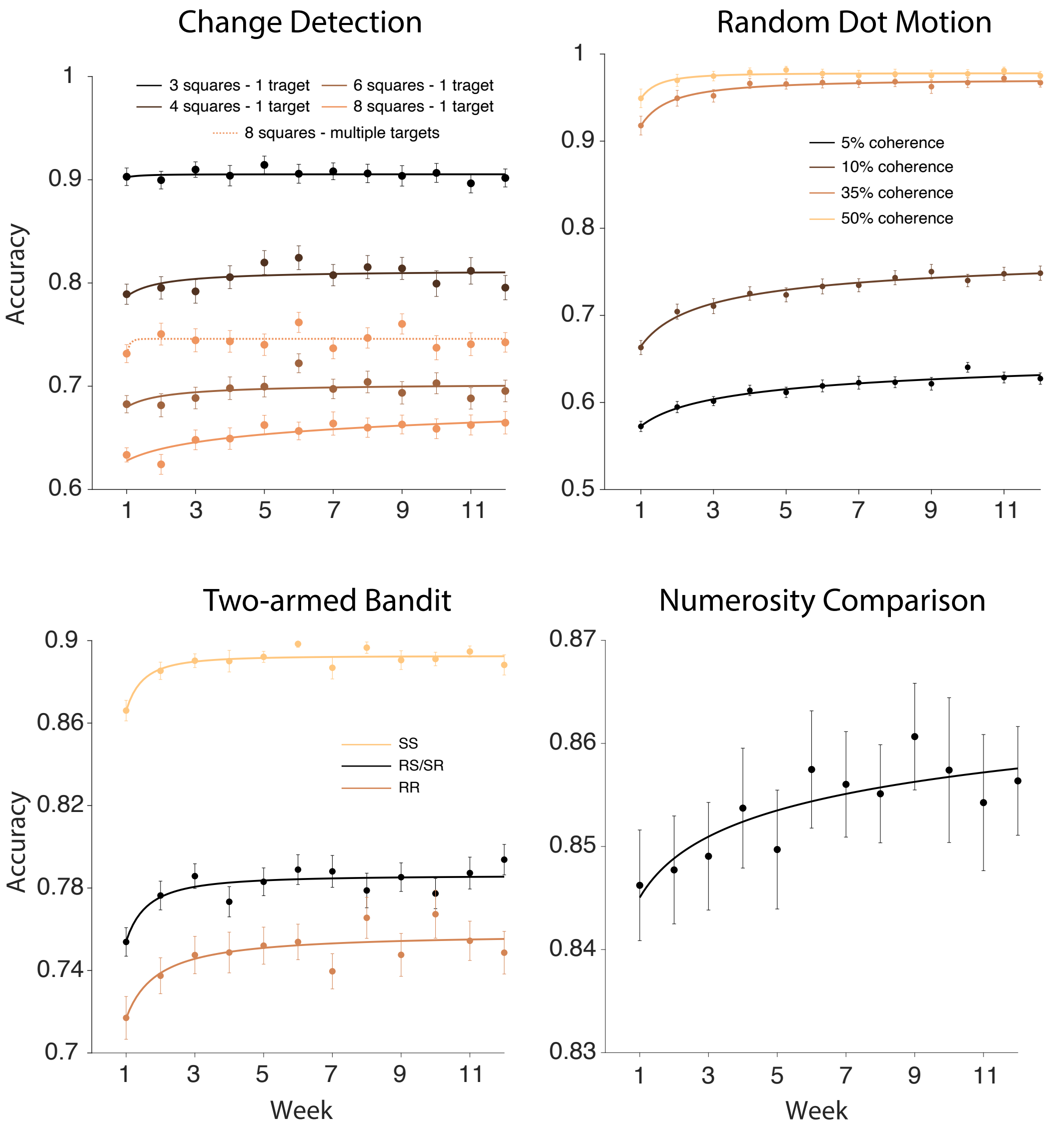

Figure S6: **Task performance over time**. For each task we calculated participants' accuracy throughout the study. Since the Intertemporal Choice and Lottery Ticket tasks had no objective performance measure, we do not plot behavioral data for these two tasks. Dots represent mean performance across participants in each week ± s.e.m..

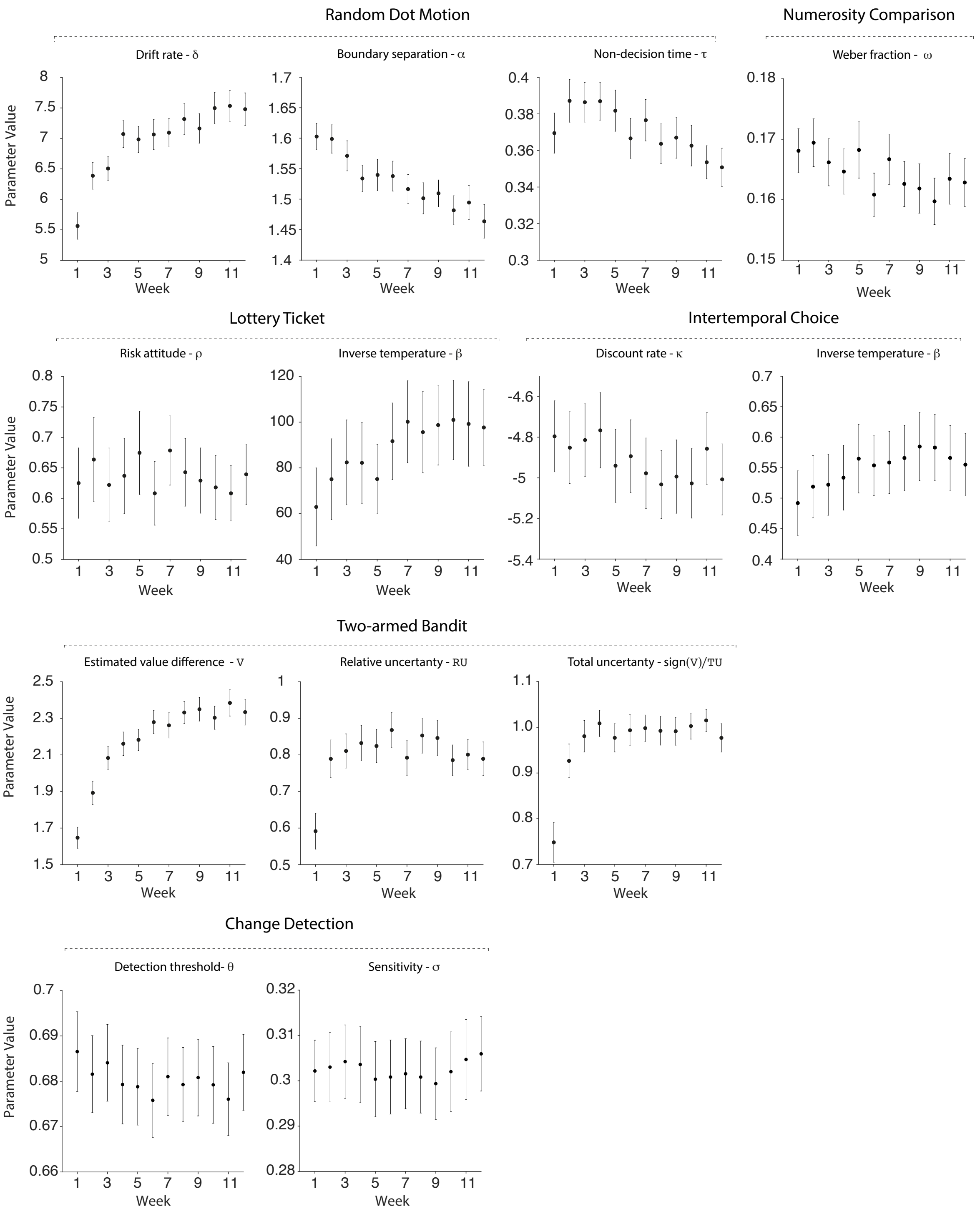

Figure S7: **The dynamic computational phenotype**. For each parameter in each task, we plotted the mean parameter value (derived from the independent model) across time. Dots represent mean parameter values across participants in each week ± s.e.m..

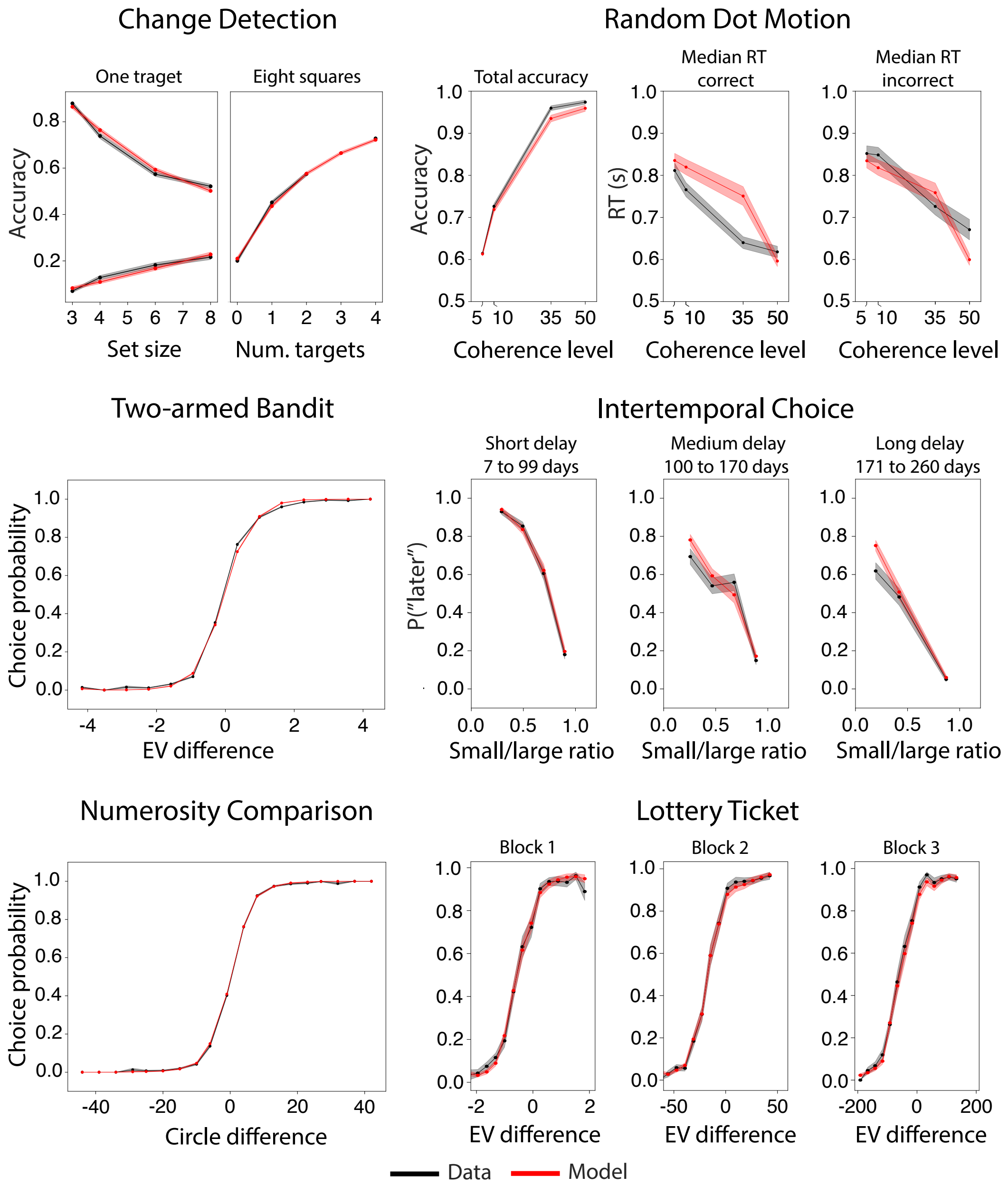

Figure S8: **Posterior predictive checks using the independent models**. We used the tasks parameters estimated using the dynamic model to predict participant's behavior in all tasks. As can be seen from the figure, the dynamic models captured well participants' real behavior. Model fit is presented in red and participants' behavior is presented in black. Shaded areas show s.e.m. across participants. See Figure 2 in the main text for posterior predictive checks for the Go/No-Go task.

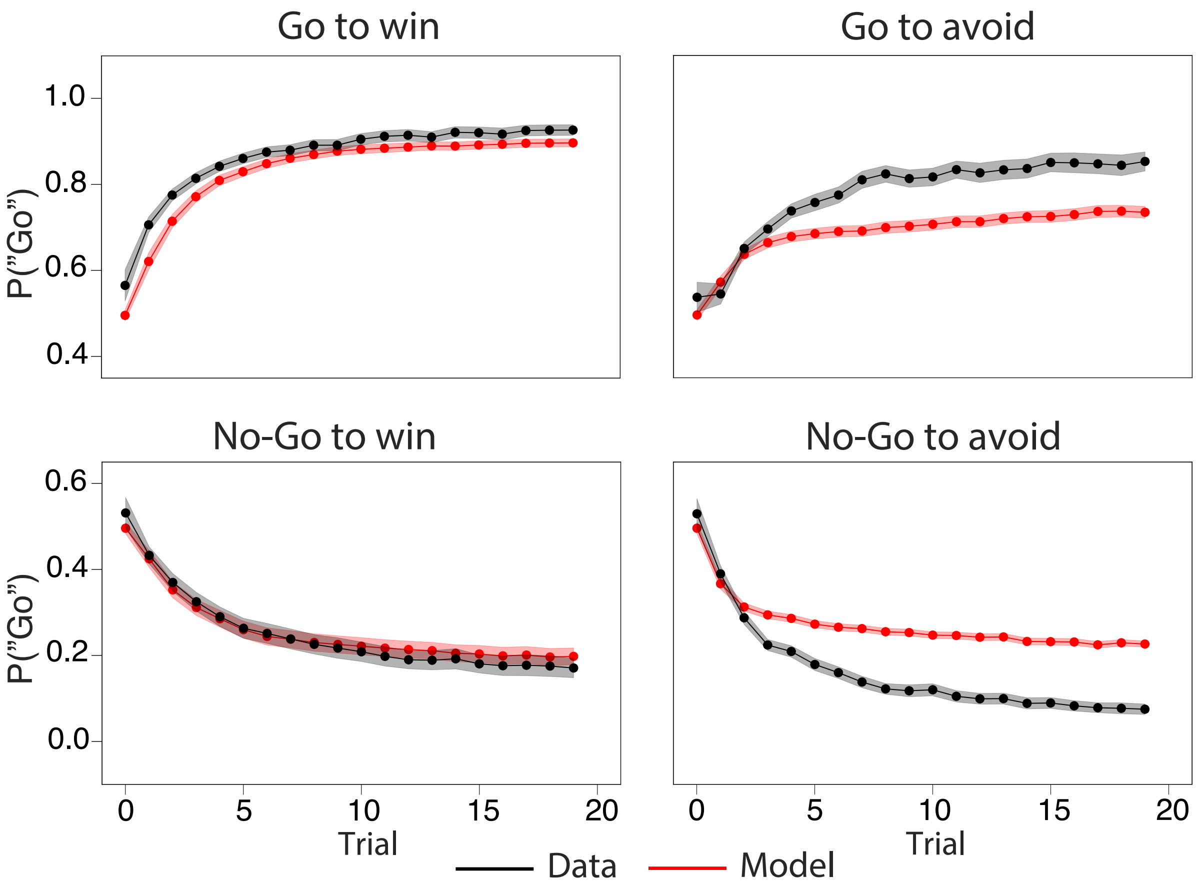

Figure S9: **Posterior predictive checks using a misspecified Go/No-Go model**. We used the parameter estimates from the independent model using the original formulation described in [9] to predict the probability of taking a "Go" action in different task conditions. As can be seen from the figure, this model failed to capture participants' behavior in conditions that required action avoidance ("Go to avoid" and "No-Go to avoid"). Model fit is presented in red and participants' behavior is presented in black. Shaded areas show s.e.m. across participants.

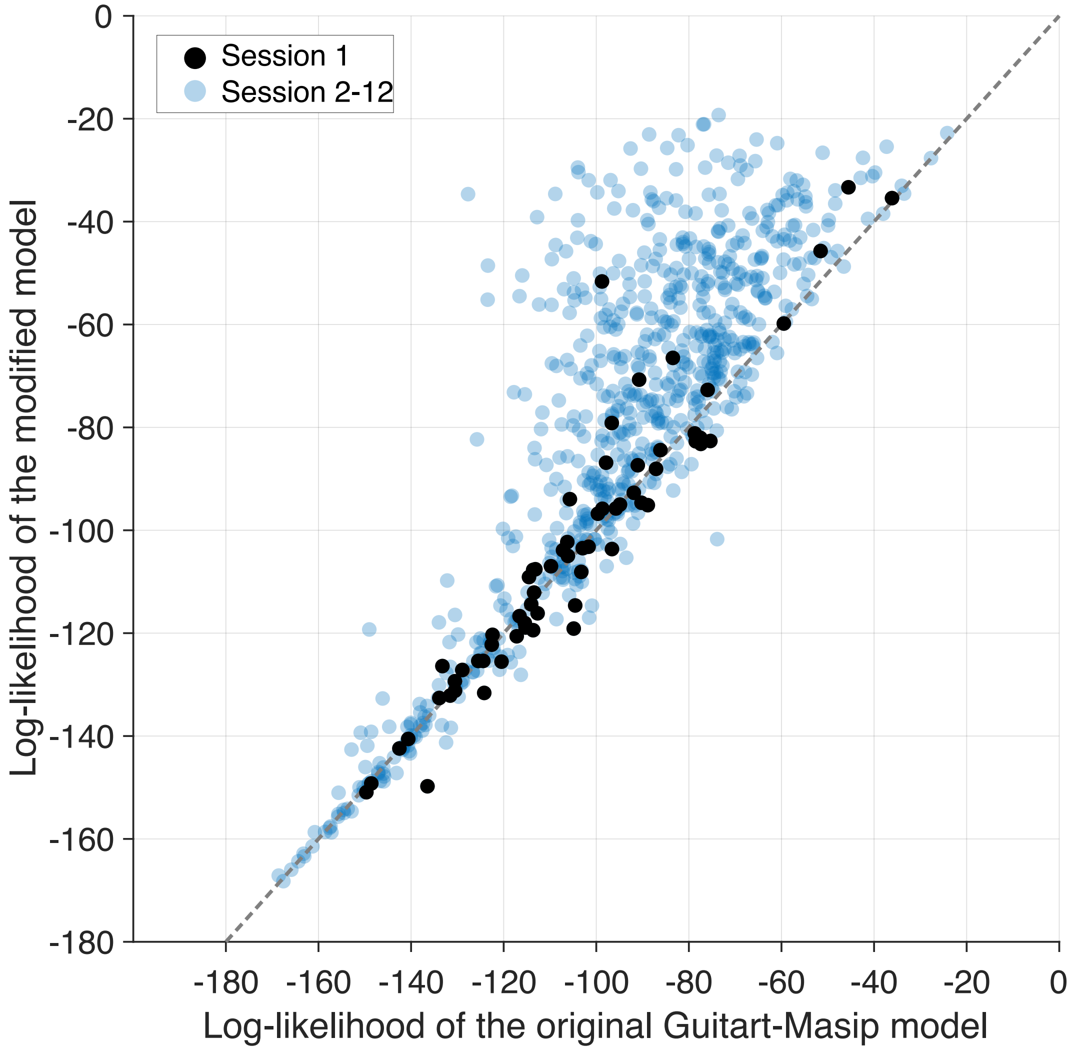

Figure S10: **Log-likelihood of the misspecified and modified Go/No-Go models**. We fitted the misspecified and modified Go/No-Go models to the behavioral data from session 1 (dark dots) and from rest of the sessions (2-12; bright dots). As can be seen from the figure, for sessions 2-12, the modified model resulted in higher log-likelihood (meaning it fit the data better) compared with the misspecified model. No difference in model fit was observed for session 1.

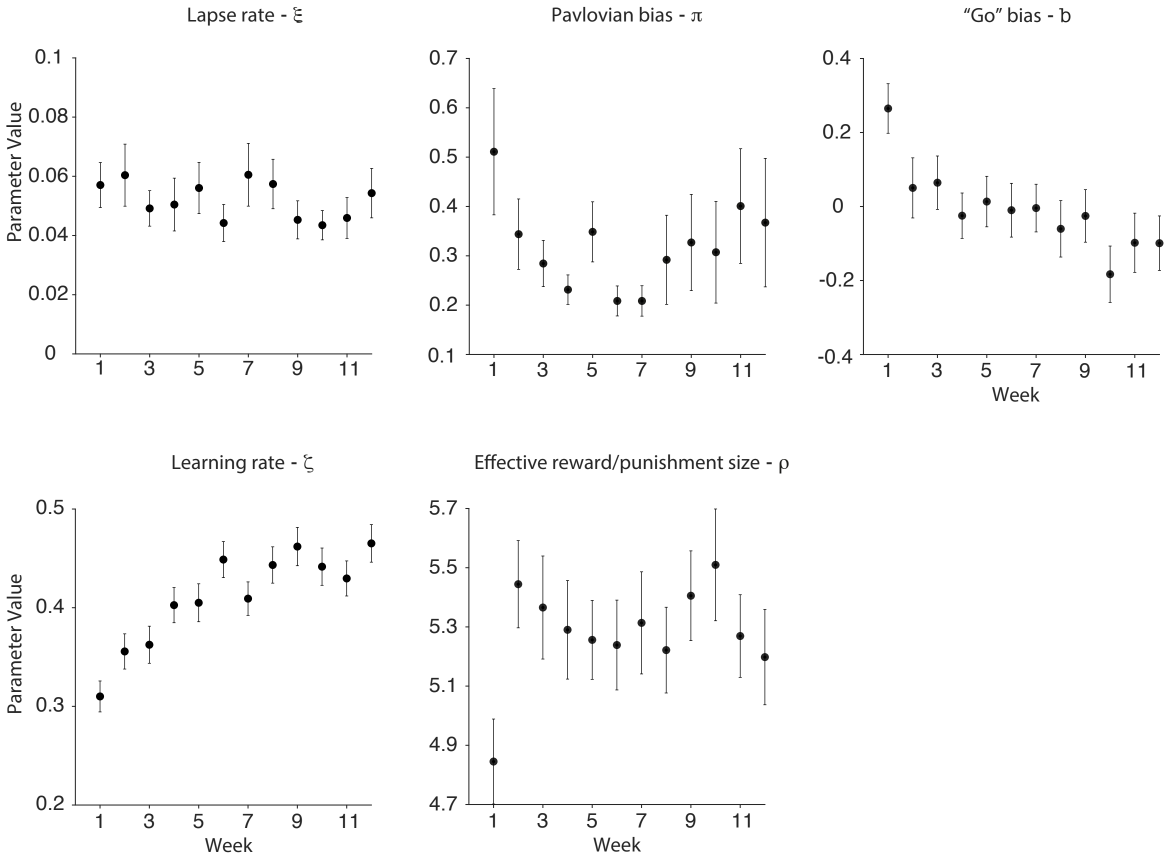

Figure S11: **The dynamic computational phenotype using a misspecified Go/No-Go model**. For each parameter, we show its mean and standard error across participants in each week (derived from the independent model).

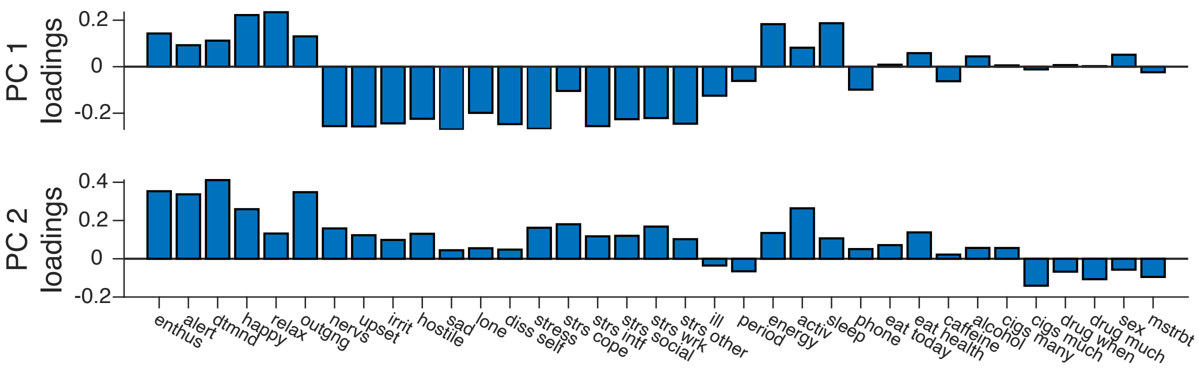

Figure S12: **Loadings of the daily survey questions**. For each survey question we plot the loading values in each of the two factors. Top: PC1 - Affective valence. Bottom: PC2 - Affective arousal.

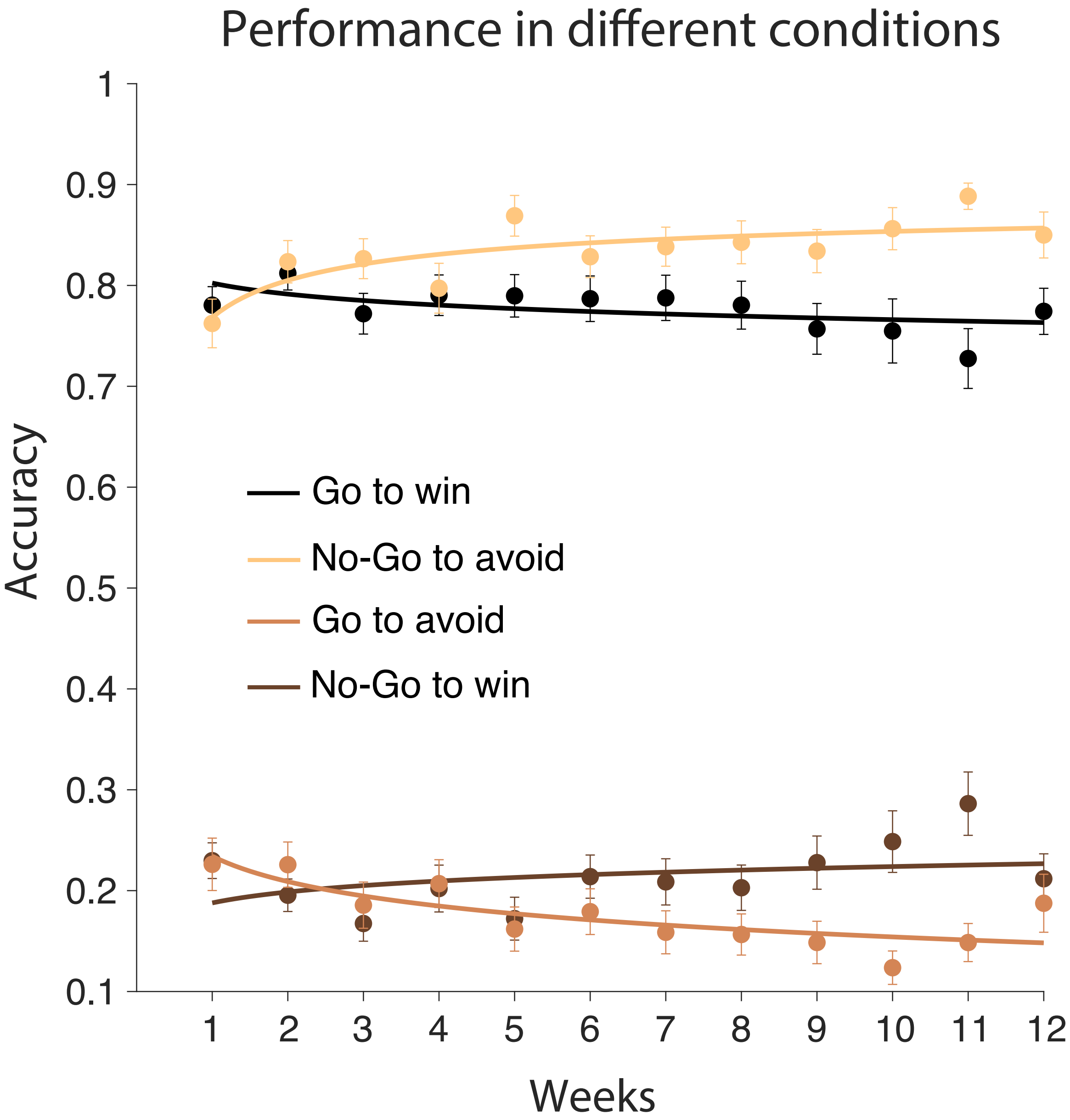

Figure S13: **Behavioral performance of excluded participants in the Go/No-Go task**. While the non-learners' performance in "Go to win" and "No-Go to avoid" conditions was comparable with the performance of learners, non-learners performed much wrose in the "Go to avoid" and "No-Go to win" conditions. Compare to Figure 3a in the main text.

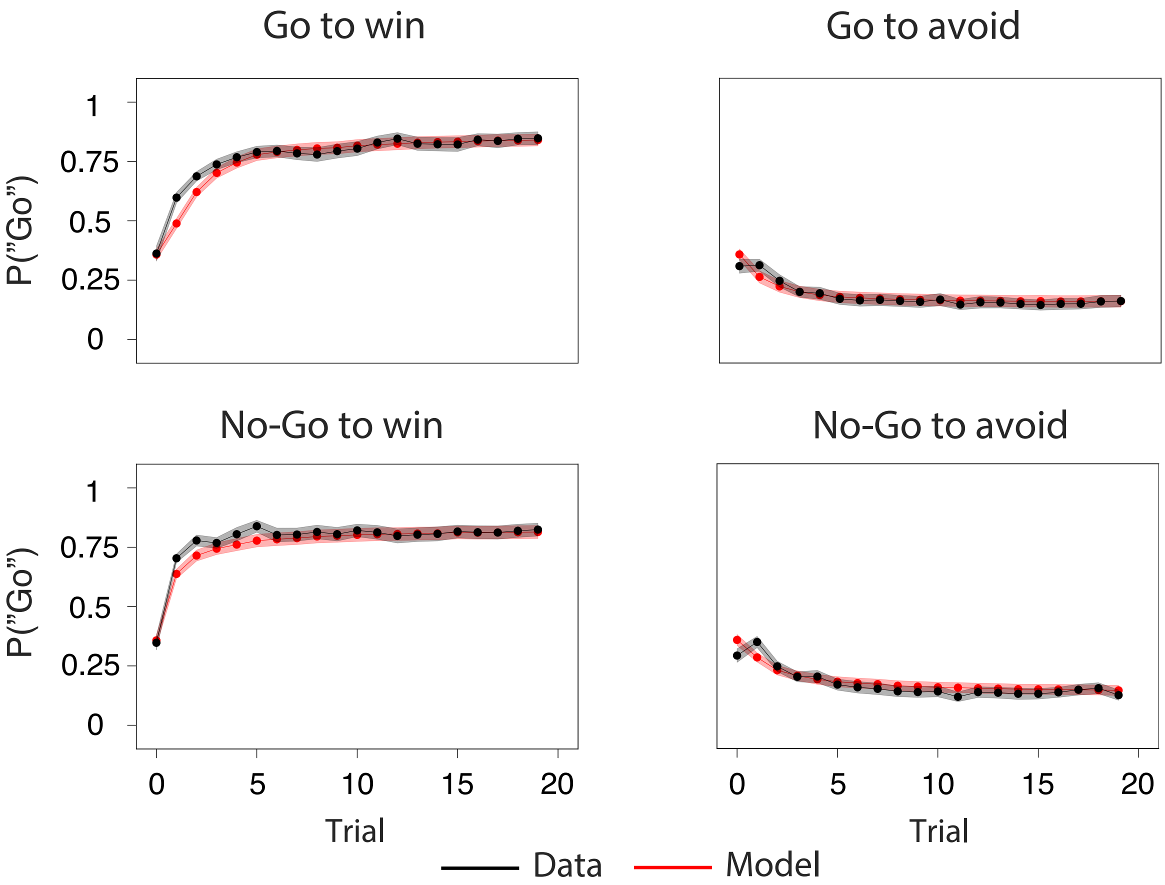

Figure S14: **Posterior predictive checks of excluded participants in the Go/No-Go task**. We used the parameter estimates from the independent model to predict the probability of taking a "Go" action in different task conditions. Model fits (red) closely matched behavior (black), however note the dramatic differences in the probability to "Go" in the "Go to avoid" and "No-Go to win" conditions compared with "learners". Real and predicted data were averaged across blocks and weeks for each participant. Shaded areas show standard error of the mean (s.e.m.) across participants. Compare to Figure 3b in the main text.

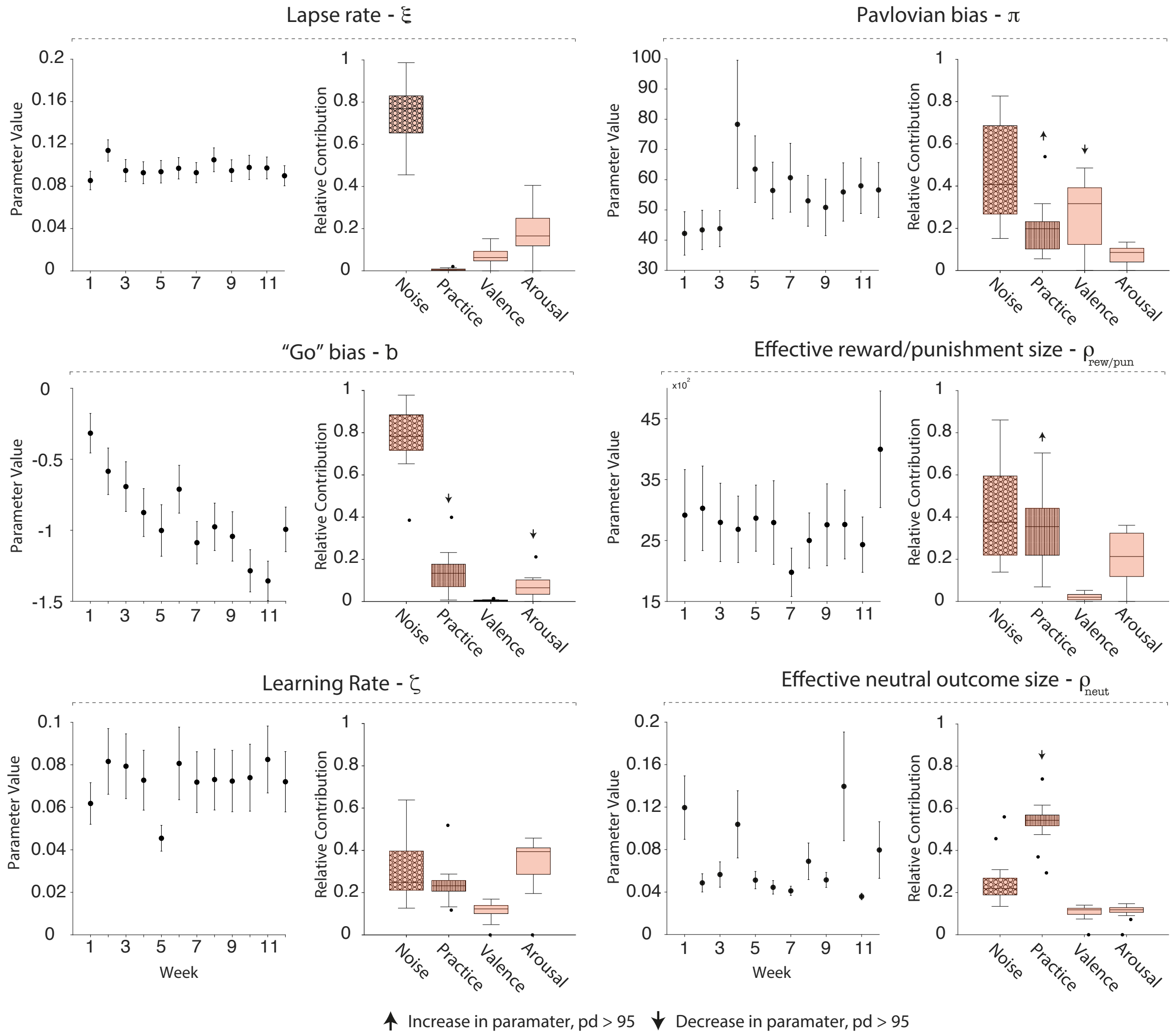

Figure S15: **The dynamic computational phenotype in the Go/No-Go task for non-learners**. For each parameter estimated for non-learners, the left plot shows its mean and standard error across participants in each week (derived from the independent model), and the right plot shows the relative contribution of each source (derived from the dynamic model). Arrows indicate the direction of the effect on each parameter over time - positive (up) or negative (down), for effects with pd > 95. Error bars show standard error of the mean (s.e.m.) across participants. Compare to Figure 4 in the main text; note the difference in parameter scales.

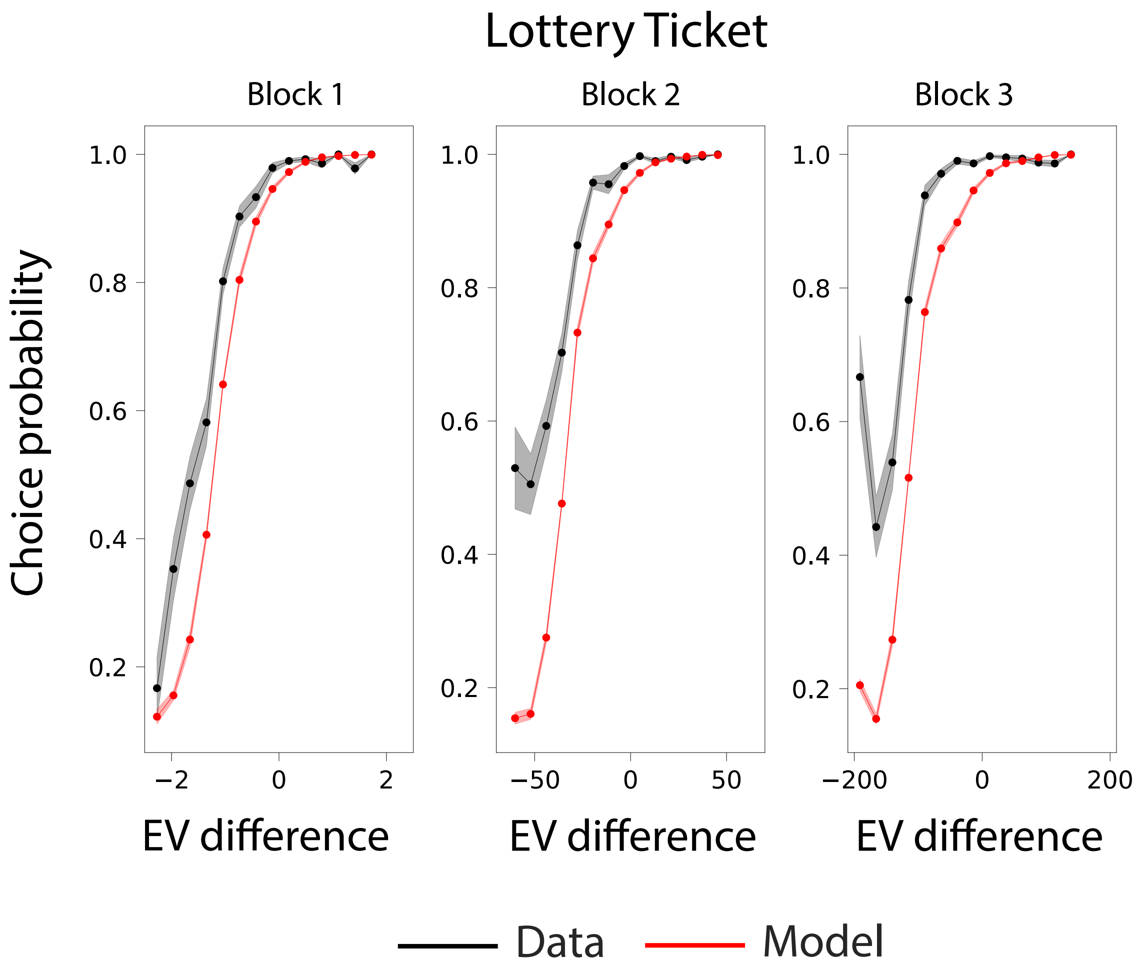

Figure S16: **Posterior predictive checks of excluded participants in the Lottery ticket task task**. We used the parameter estimates from the independent model to predict the choice probability in different task blocks. Model fits (red) matched behavior (black) comparably well only in the Block 1, where the EV differences was small. Real and predicted data were averaged across participants and weeks. Shaded areas show standard error of the mean (s.e.m.) across participants. Compare to posterior predictive checks for this task presented in Supplementary Figure S8.

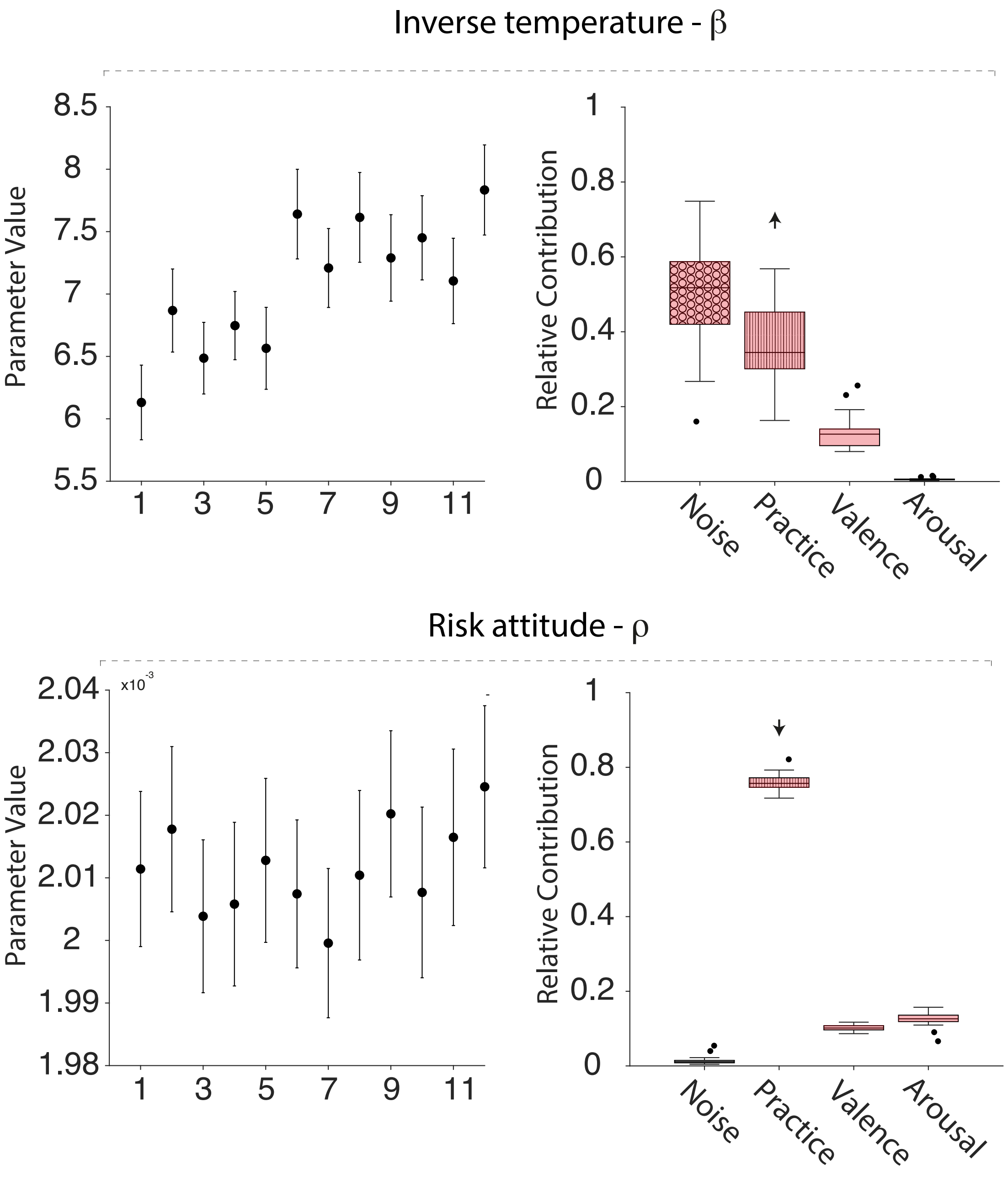

Figure S17: **The dynamic computational phenotype in the Lottery ticket task for the excluded participants**. For each parameter estimated for the excluded participants, the left plot shows its mean and standard error across participants in each week (derived from the independent model), and the right plot shows the relative contribution of each source (derived from the dynamic model). Arrows indicate the direction of the effect on each parameter over time - positive (up) or negative (down), for effects with pd > 95. Error bars show standard error of the mean (s.e.m.) across participants. Compare to Figure 5 in the main text and Supplementary Figure S7; note the difference in parameter scales.

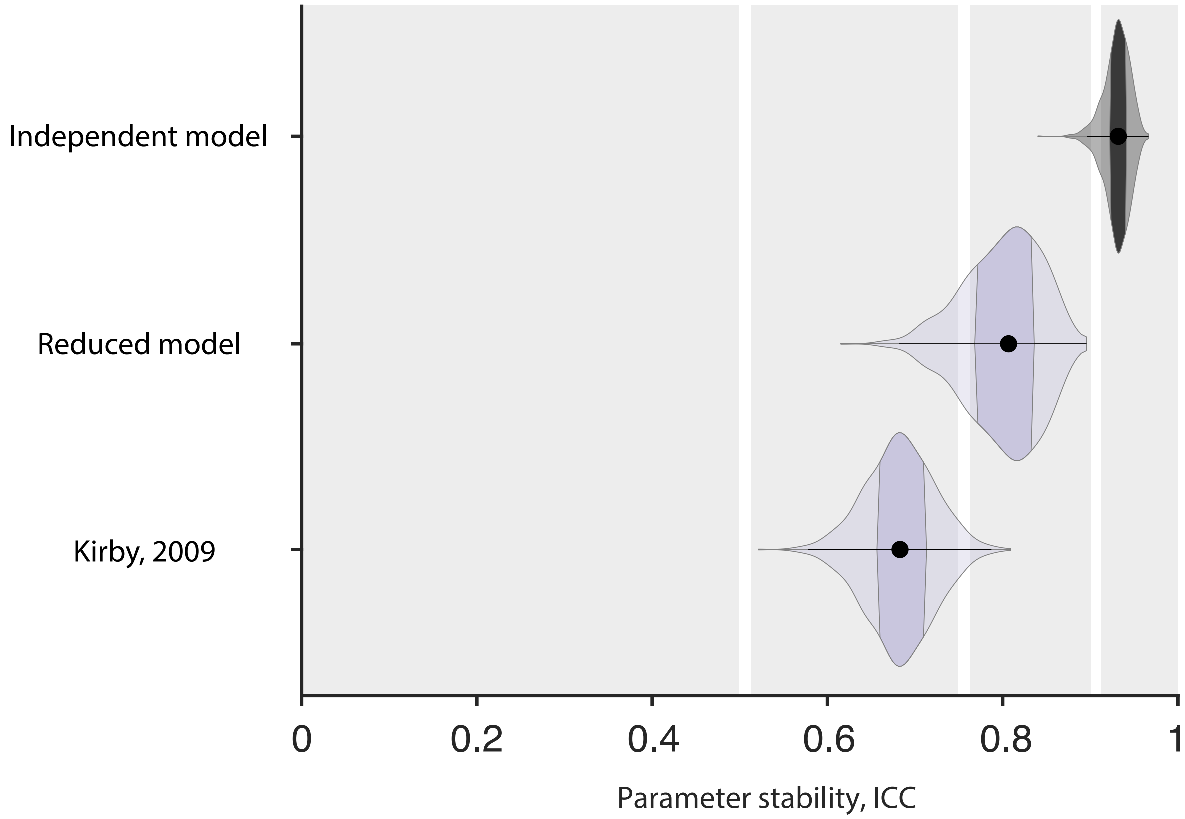

Figure S18: **Stability of the discount rate parameter under different computational models**. Violin plots show the bootstrapped stability estimates in terms of intraclass correlations (ICC) for the discount parameter (1000 bootstrap iterations over participants; viloin colors indicate tasks). We used three methods to estimate the discount rate parameter - the indemendent model, the reduced model, and the original hyperbolic function without softmax decision rule. Points mark the median, shaded areas mark the inter-quartile range. As can be seen from the figure, using the hyperbolic function without softmax decision rule and without hierarchical modeling resulted in lower parameter stability. Gray regions mark a common interpretation of ICC values [23]: < 0.5 (poor), $0.5 - 0.75$ (moderate), $0.75 - 0.9$ (good), and > 0.9 (excellent).

References

[1] Patrick Wilken and Wei Ji Ma. A detection theory account of change detection. *Journal of Vision*, 4(12):11–11, 2004. doi:https://doi.org/10.1167/4.12.11.

[2] Sivananda Rajananda, HW Lau, and Brian Odegaard. A random-dot kinematogram for web-based vision research. *Journal of Open Research Software*, 6(1), 2018. doi:10.5334/jors.194.

[3] Veronika Lerche, Andreas Voss, and Markus Nagler. How many trials are required for parameter estimation in diffusion modeling? A comparison of different optimization criteria. *Behavior Research Methods*, 49(2):513–537, 2017. doi:https://doi.org/10.3758/s13428-016-0740-2.

[4] Roger Ratcliff and Gail McKoon. The diffusion decision model: theory and data for two-choice decision tasks. *Neural Computation*, 20:873–922, 2008. doi:10.1162/neco.2008.12-06-420.

[5] George Ainslie. Specious reward: A behavioral theory of impulsiveness and impulse control. *Psychological Bulletin*, 82(4):463, 1975. doi:https://doi.org/10.1037/h0076860.

[6] Howard Rachlin, Jay Brown, and David Cross. Discounting in judgments of delay and probability. *Journal of Behavioral Decision Making*, 13(2):145–159, 2000. doi:https://doi.org/10.1002/(SICI)1099-0771(200004/06)13:2<145::AID-BDM320>3.0.CO;2-4.

[7] Kris N Kirby. One-year temporal stability of delay-discount rates. *Psychonomic Bulletin & Review*, 16(3):457–462, 2009. doi:https://doi.org/10.3758/PBR.16.3.457.

[8] James E. Mazur. An adjusting procedure for studying delayed reinforcement. In Michael L. Commons, James E. Mazur, John A. Nevin, and Howard Rachlin, editors, *Quantitative analyses of behavior: The effect of delay and of intervening events on reinforcement value*, volume 5, pages 55–73. Erlbaum, Hillsdale, NJ, 1987.

[9] Marc Guitart-Masip, Quentin J.M. Huys, Lluis Fuentemilla, Peter Dayan, Emrah Duzel, and Raymond J. Dolan. Go and no-go learning in reward and punishment: Interactions between affect and effect. *NeuroImage*, 62(1):154–166, 2012. doi:10.1016/j.neuroimage.2012.04.024.

[10] Stefano Palminteri, Mehdi Khamassi, Mateus Joffily, and Giorgio Coricelli. Contextual modulation of value signals in reward and punishment learning. *Nature Communications*, 6(1):8096, 2015. doi:https://doi.org/10.1038/ncomms9096.

[11] Stefano Palminteri and Maël Lebreton. Context-dependent outcome encoding in human reinforcement learning. *Current Opinion in Behavioral Sciences*, 41:144–151, 2021. doi:https://doi.org/10.1016/j.cobeha.2021.06.006.

[12] Roger Ratcliff. A theory of memory retrieval. *Psychological Review*, 85(2):59, 1978. doi:https://doi.org/10.1037/0033-295X.85.2.59.

[13] Charles A. Holt and Susan K. Laury. Risk aversion and incentive effects. *American Economic Review*, 92(5):1644–1655, 2002. doi:10.1257/000282802762024700.

[14] Sebastian Olschewski, Pavel Sirotkin, and Jörg Rieskamp. Empirical underidentification in estimating random utility models: The role of choice sets and standardizations. *British Journal of Mathematical and Statistical Psychology*, 75(2):252–292, 2022. doi:https://doi.org/10.1111/bmsp.12256.

[15] Samuel J. Gershman. Uncertainty and exploration. *Decision*, 6(3):277–286, 2019. doi:10.1037/dec0000101.

[16] David J. Getty. Discrimination of short temporal intervals: A comparison of two models. *Perception & Psychophysics*, 18(1):1–8, 1975. doi:https://doi.org/10.3758/BF03199358.

[17] Holly Sullivan-Toole, Nathaniel Haines, Kristina Dale, and Thomas M. Olino. Enhancing the psychometric properties of the iowa gambling task using full generative modeling. *Computational Psychiatry*, 6(1):189, 2022. doi:10.5334/cpsy.89.

[18] Maria Waltmann, Florian Schlagenhauf, and Lorenz Deserno. Sufficient reliability of the behavioral and computational readouts of a probabilistic reversal learning task. *Behavior Research Methods*, 54(6):2993–3014, 2022. doi:https://doi.org/10.3758/s13428-021-01739-7.

[19] Kentaro Katahira. How hierarchical models improve point estimates of model parameters at the individual level. *Journal of Mathematical Psychology*, 73:37–58, 2016. doi:10.1016/j.jmp.2016.03.007.

[20] Craig Hedge, Georgina Powell, and Petroc Sumner. The reliability paradox: Why robust cognitive tasks do not produce reliable individual differences. *Behavior Research Methods*, 50(3):1166–1186, Jun 2018. doi:10.3758/s13428-017-0935-1.

[21] Kenneth O. McGraw and Seok P. Wong. Forming inferences about some intraclass correlation coefficients. *Psychological Methods*, 1(1):30–46, 1996. doi:10.1037/1082-989X.1.1.30.

[22] Aki Vehtari, Andrew Gelman, Daniel Simpson, Bob Carpenter, and Paul-Christian Bürkner. Rank-normalization, folding, and localization: An improved R^2 for assessing convergence of MCMC (with discussion). *Bayesian Analysis*, 16(2):667–718, 2021. doi:10.1214/20-BA1221.

[23] Terry K. Koo and Mae Y. Li. A guideline of selecting and reporting intraclass correlation coefficients for reliability research. *Journal of Chiropractic Medicine*, 15(2):155–163, 2016. Erratum in: Journal of Chiropractic Medicine, 16(4):346. doi:10.1016/j.jcm.2016.02.012.
