# Supplementary material for: Dynamic computational phenotyping of human cognition
Source: Nat Hum Behav. 2024 Feb 8;8(5):917–31. doi: 10.1038/s41562-024-01814-x (PMC11132988; doi:10.1038/s41562-024-01814-x)
Supplement: Supplementary file 2 — Reporting Summary [file 41562_2024_1814_MOESM2_ESM.pdf]

Corresponding author(s): Roey Schurr, PhD, Daniel Reznik, PhD

Last updated by author(s): Dec 12, 2023

Reporting Summary

Nature Portfolio wishes to improve the reproducibility of the work that we publish. This form provides structure for consistency and transparency in reporting. For further information on Nature Portfolio policies, see our Editorial Policies and the Editorial Policy Checklist.

Statistics

For all statistical analyses, confirm that the following items are present in the figure legend, table legend, main text, or Methods section.

n/a	Confirmed	
☐	☒	The exact sample size (*n*) for each experimental group/condition, given as a discrete number and unit of measurement
☐	☒	A statement on whether measurements were taken from distinct samples or whether the same sample was measured repeatedly
☐	☒	The statistical test(s) used AND whether they are one- or two-sided<br>*Only common tests should be described solely by name; describe more complex techniques in the Methods section.*
☐	☒	A description of all covariates tested
☒	☐	A description of any assumptions or corrections, such as tests of normality and adjustment for multiple comparisons
☐	☒	A full description of the statistical parameters including central tendency (e.g. means) or other basic estimates (e.g. regression coefficient) AND variation (e.g. standard deviation) or associated estimates of uncertainty (e.g. confidence intervals)
☒	☐	For null hypothesis testing, the test statistic (e.g. *F*, *t*, *r*) with confidence intervals, effect sizes, degrees of freedom and *P* value noted<br>*Give P values as exact values whenever suitable.*
☐	☒	For Bayesian analysis, information on the choice of priors and Markov chain Monte Carlo settings
☐	☒	For hierarchical and complex designs, identification of the appropriate level for tests and full reporting of outcomes
☒	☐	Estimates of effect sizes (e.g. Cohen's *d*, Pearson's *r*), indicating how they were calculated

Our web collection on statistics for biologists contains articles on many of the points above.

Software and code

Policy information about availability of computer code

Data collection	Amazon Mechanical Turk
Data analysis	Python 3.9.0, MATLAB 2019b, R 4.2.3, Stan 2.27, CmdStanPy 0.9.77.<br><br>Code availability<br>The custom code used in this study is freely available at https://github.com/roeysc/dynamic_computational_phenotyping/tree/main/code.

For manuscripts utilizing custom algorithms or software that are central to the research but not yet described in published literature, software must be made available to editors and reviewers. We strongly encourage code deposition in a community repository (e.g. GitHub). See the Nature Portfolio guidelines for submitting code & software for further information.

Data

Policy information about availability of data

All manuscripts must include a data availability statement. This statement should provide the following information, where applicable:

- Accession codes, unique identifiers, or web links for publicly available datasets
- A description of any restrictions on data availability
- For clinical datasets or third party data, please ensure that the statement adheres to our policy

Data availability
The data used in this study are freely available at https://github.com/roeysc/dynamic_computational_phenotyping/tree/main/data.

Research involving human participants, their data, or biological material

Policy information about studies with human participants or human data. See also policy information about sex, gender (identity/presentation), and sexual orientation and race, ethnicity and racism.

Reporting on sex and gender	Out of the participants that were included in the analyses, 47 identified themselves as males, 41 identified as females, 1 identified as queer. No sex/gender-related hypotheses were tested in the current study as we considered groups effects only. Future studies can potentially explore whether the effects reported in the current study differ between groups.
Reporting on race, ethnicity, or other socially relevant groupings	No socially relevant categorization variables were used in the current study.
Population characteristics	We recruited adult (>18 years old) Amazon Mechanical Turk participants registered in the USA. The participant pool was filtered to have an approval rating of 90% or above In order to increase the likelihood for continued participation throughout the study. More detailed information on the demographics of Amazon Mechanical Turk participants pool can be found in https://crowdsourcing-class.org/readings/downloads/platform/demographics-of-mturk.pdf .
Recruitment	Participants were recruited from Amazon Mechanical Turk through CloudResearch services. Due to the longitudinal nature of our study, participants were rewarded with monetary bonuses after completing half of the study ($5) and the entire study ($10). Therefore, it is possible that our participants were the ones who are particularly interested in increasing their compensation. However, since our rigid selection criterion was approval rating of 90% or above, we do not believe that this monetary-based potential self-selection bias influenced participants' task performance or affected our results.
Ethics oversight	Participants read the study description via Amazon Mechanical Turk and indicated whether they agree to participate with a button press. The study was approved by the Institutional Review Board of Harvard University and was performed in accordance with the relevant guidelines and regulations.

Note that full information on the approval of the study protocol must also be provided in the manuscript.

Field-specific reporting

Please select the one below that is the best fit for your research. If you are not sure, read the appropriate sections before making your selection.

☐ Life sciences ☒ Behavioural & social sciences ☐ Ecological, evolutionary & environmental sciences

For a reference copy of the document with all sections, see nature.com/documents/nr-reporting-summary-flat.pdf

Behavioural & social sciences study design

All studies must disclose on these points even when the disclosure is negative.

Study description	We performed a quantitative study in which human participants performed computer-based tasks.
Research sample	Participants were recruited from Amazon Mechanical Turk through CloudResearch services. Our longitudinal design involved multi-day testing each week (during 12 weeks). Therefore we recruited participants online to allow for temporal flexibility. Ninety participants were included in the final analysis (mean age: 39.4 ± 10.8 years; 47 identified as males, 41 identified as females, 1 identified as queer). The male/female ratio in our sample was nearly 0.5; the mean age of USA residents in 2020 was 38.6 (www.statista.com), which is only slightly lower than the mean age in the current sample.
Sampling strategy	The sample size (N=90 participants) was determined based on the size of samples often used in the literature. As shown in a literature review by Karvelis et al. 2022 (“Individual Differences in Computational Psychiatry: A Review of Current Challenges”), most behavioral studies that calculated test-retest reliability of the computational phenotype used 30-60 participants. Since retention in longitudinal studies is often a concern, we recruited 141 participants and ended up with 90 participants who completed the study. We stress here that fitting model parameters using a hierarchical Bayesian framework pools information both across participants and across time (in this longitudinal design). Therefore, we expected a sample size of 90 participants to be more than enough for our

	purposes. As for the sampling strategy, participants were sampled randomly. In other words, we did not use any specific strategy to sample participants through the online platform.
Data collection	Participants were recruited from Amazon Mechanical Turk through CloudResearch services and performed all the tasks using their personal computers in their own time, given study limitations. There were no experimental groups in the study and all participants followed the same experimental procedure. Researchers were blind to the study hypotheses during data collection.
Timing	Data collection started in December 1st 2019 and ended in May 27 2020.
Data exclusions	We only included participants who had at least 6 weeks of task data with no more than three missing consecutive weeks for each task.
Non-participation	Out of the initial cohort of 141 participants, 90 participants satisfied these criteria.
Randomization	Participants were not allocated into experimental groups.

Reporting for specific materials, systems and methods

We require information from authors about some types of materials, experimental systems and methods used in many studies. Here, indicate whether each material, system or method listed is relevant to your study. If you are not sure if a list item applies to your research, read the appropriate section before selecting a response.

Materials & experimental systems

n/a	Involved in the study
☒	☐ Antibodies
☒	☐ Eukaryotic cell lines
☒	☐ Palaeontology and archaeology
☒	☐ Animals and other organisms
☒	☐ Clinical data
☒	☐ Dual use research of concern
☒	☐ Plants

Methods

n/a	Involved in the study
☒	☐ ChIP-seq
☒	☐ Flow cytometry
☒	☐ MRI-based neuroimaging

Plants

Seed stocks	NA
Novel plant genotypes	NA
Authentication	NA
